# An extensional strain sensing mechanosome drives adhesion-independent platelet activation at supraphysiological hemodynamic gradients

**DOI:** 10.1186/s12915-022-01274-7

**Published:** 2022-03-24

**Authors:** Nurul A. Zainal Abidin, Eric K. W. Poon, Crispin Szydzik, Mariia Timofeeva, Farzan Akbaridoust, Rose J. Brazilek, Francisco J. Tovar Lopez, Xiao Ma, Chitrarth Lav, Ivan Marusic, Philip E. Thompson, Arnan Mitchell, Andrew S. H. Ooi, Justin R. Hamilton, Warwick S. Nesbitt

**Affiliations:** 1 The Australian Centre for Blood Diseases, Monash University, Melbourne, VIC 3004 Australia; 2 Department of Medicine, St Vincent’s Hospital, Melbourne Medical School, Faculty of Medicine, Dentistry & Health Sciences, The University of Melbourne, Fitzroy, VIC 3065 Australia; 3 School of Engineering, RMIT University, La Trobe Street, Melbourne, VIC 3004 Australia; 4 Department of Mechanical Engineering, Faculty of Engineering and Information Technology, The University of Melbourne, Melbourne, VIC 3010 Australia; 5 Medicinal Chemistry, Monash Institute of Pharmaceutical Sciences, Monash University, Parkville, VIC 3052 Australia; 6 CFD Methodology Group, Scuderia AlphaTauri F1, Bicester, OX26 4LD UK

**Keywords:** Platelet, Hemodynamics, Mechanotransduction, Extensional strain

## Abstract

**Background:**

Supraphysiological hemodynamics are a recognized driver of platelet activation and thrombosis at high-grade stenosis and in blood contacting circulatory support devices. However, whether platelets mechano-sense hemodynamic parameters directly in free flow (in the absence of adhesion receptor engagement), the specific hemodynamic parameters at play, the precise timing of activation, and the signaling mechanism(s) involved remain poorly elucidated.

**Results:**

Using a generalized Newtonian computational model in combination with microfluidic models of flow acceleration and quasi-homogenous extensional strain, we demonstrate that platelets directly mechano-sense acute changes in free-flow extensional strain independent of shear strain, platelet amplification loops, von Willebrand factor, and canonical adhesion receptor engagement. We define an extensional strain sensing “mechanosome” in platelets involving cooperative Ca^2+^ signaling driven by the mechanosensitive channel Piezo1 (as the primary strain sensor) and the fast ATP gated channel P2X1 (as the secondary signal amplifier). We demonstrate that type II PI3 kinase C2α activity (acting as a “clutch”) couples extensional strain to the mechanosome.

**Conclusions:**

Our findings suggest that platelets are adapted to rapidly respond to supraphysiological extensional strain dynamics, rather than the peak magnitude of imposed wall shear stress. In the context of overall platelet activation and thrombosis, we posit that “extensional strain sensing” acts as a priming mechanism in response to threshold levels of extensional strain allowing platelets to form downstream adhesive interactions more rapidly under the limiting effects of supraphysiological hemodynamics.

**Supplementary Information:**

The online version contains supplementary material available at 10.1186/s12915-022-01274-7.

## Background

Blood platelets are anucleate cells derived from megakaryocytes that are central to physiological hemostasis and are a key player in pathological thrombosis. Mechanical hemodynamic parameters are major drivers of both physiological and pathological platelet function. At sites of arterial stenosis, associated with atherosclerotic disease, or within blood contacting mechanical circulatory support (MCS) and extracorporeal membrane oxygenation (ECMO) circuits, platelets can experience a wide array of mechanical forces that impact their function, including elevated wall strain rates ($\dot{\gamma}$) and local fluid stresses (*τ*) [1, 2], mass transport phenomena due to erythrocyte margination effects and flow recirculation [3], tensile and compressive forces upon impact and adhesion at sites of subendothelial matrix exposure and artificial device surfaces [4, 5], velocity gradients, and extensional (elongational) strain due to flow acceleration [6–8]. Platelets transduce these external mechanical stimuli into specific intracellular biochemical signals (mechanotransduction) that include, [Ca^2+^]_c_ flux [9] phosphoinositide synthesis [10, 11], cytoskeletal remodeling [12], and adhesion receptor engagement, activation, and signaling [13]. These processes drive platelet functional outputs, such as surface translocation, membrane tethering [6, 14], shape (morphology) change, shear-induced platelet aggregation (SIPA), and ultimately thrombus formation.

SIPA occurs when platelets convected in bulk flow aggregate upon contact with surface immobilized von Willebrand factor (VWF) and/or activated co-adhered platelets. $\dot{\gamma}$ is a critical hemodynamic parameter that regulates platelet capture from free flow, aggregation rate, and size [15]. We and others have demonstrated that platelet aggregation in the presence of VWF occurs within relatively low $\dot{\gamma}$ zones just downstream from sites of high $\dot{\gamma}$ [6]. Significantly, these studies demonstrated that the early stages of SIPA involve resting discoid platelets through the formation of passive membrane tether structures [6] in association with platelet GPIb/V/IX binding to shear (*τ* ≥ 3.5 to 5.0 Pa) decrypted VWF [6, 16, 17]. Initial engagement of these membrane tethers is followed by transient [Ca^2+^]_c_ flux and correlated active tether restructuring and aggregate consolidation [6]. Classical amplification loop signaling mediated by platelet and red blood cell (RBC) secretion of ATP, ADP, TXA_2_, and thrombin generation subsequently stabilize forming aggregates in the face of destabilizing high *τ* [18]. Significantly, when platelet amplification loops are inhibited or where flow-dependent advective clearance rates are high, elevated *τ* can destabilize and limit aggregate consolidation leading to embolization. While these findings indicate that stable platelet aggregation preferentially occurs in flow sheltered zones downstream from regions of flow acceleration, little to no evidence has been forthcoming as to whether platelets “mechano-sense” the initial free-flow acceleration phase, and how this may influence the rate of downstream membrane tethering, aggregation, and thrombus formation.

There is a clear knowledge gap with respect to how supraphysiological velocity gradients, as seen at severe stenosis or due to MCS/ECMO pump operation and fluidic circuits, impact platelet activation dynamics and functional behavior. To that end, and to isolate free-flow velocity effects from adhesion-mediated platelet activation events, we developed and employed a generalized Newtonian computational fluid dynamics (CFD)-directed microfluidics and time-averaged [Ca^2+^]_c_ imaging approach to investigate the effects of supraphysiological free-flow acceleration on platelet function. We identify a critical role for extensional strain at flow acceleration in directly modulating platelet mechanotransduction in the absence of adhesion receptor engagement. Through a series of pharmacologic inhibition and activation studies, we identify a novel “extensional strain sensing mechanosome” that primes platelets for immediate downstream platelet aggregation at supraphysiological velocity gradients.

## Results

### Platelets exhibit [Ca^2+^]_c_ transients in response to supraphysiological flow acceleration independent of canonical adhesion and agonist-dependent signaling

**Fig. 1:**
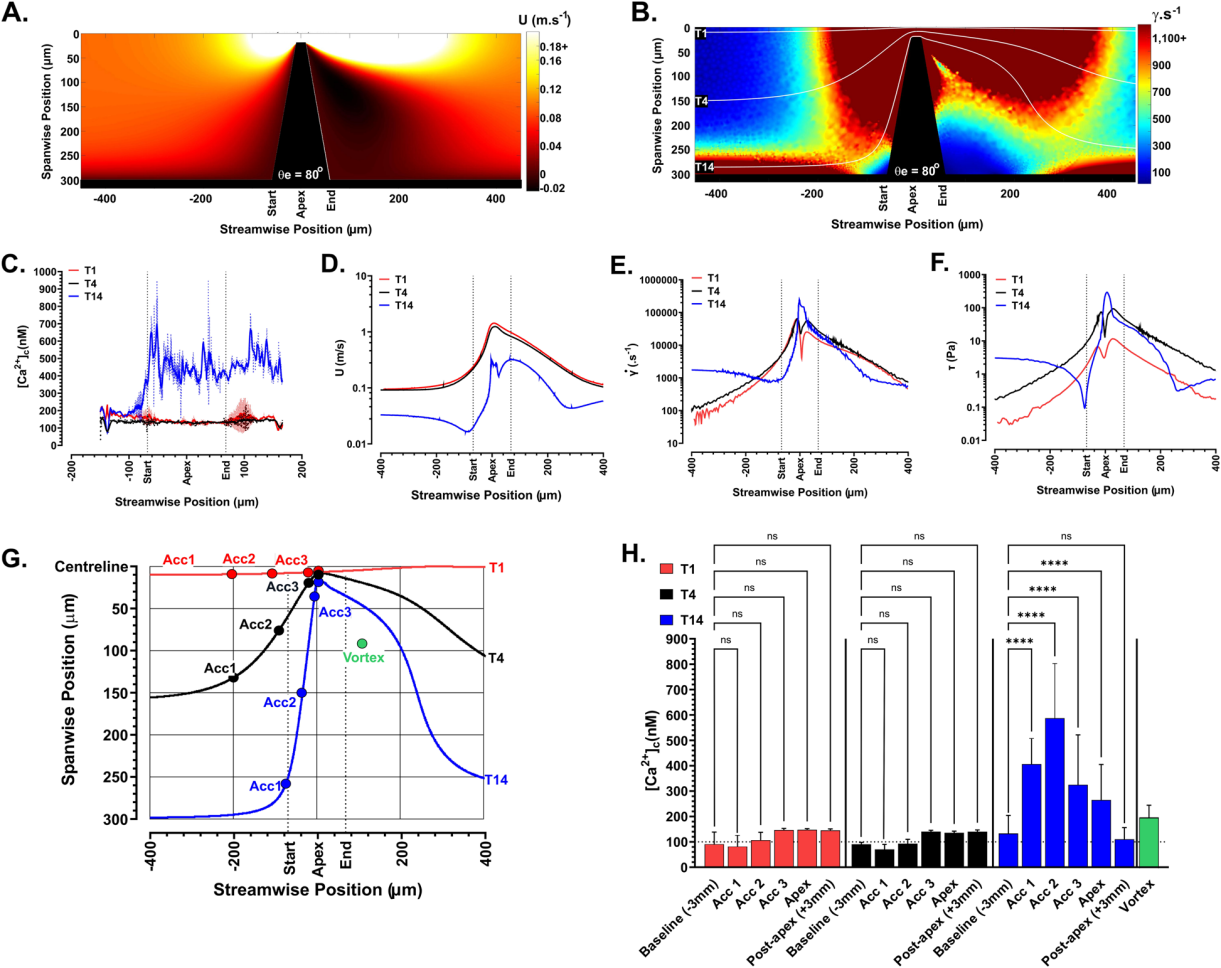
Platelet [Ca^2+^]_c_ activation is dependent on free-flow trajectory and occurs at initial flow acceleration at stenosis entry. **A**, **B** Color maps showing velocity (U m s^−1^) and strain rate ($\dot{\gamma}$ s^−1^) distributions within an *x*,*y* plane 50 μm from the microchannel floor (derived from CFD modelling of blood flow; *Q* = 200 μL/min) at a prototypical stenosis with *θ*_e_ = 80°. White lines on the strain rate map show T1-, T4-, and T14-predicted platelet trajectories (2 μm particles) (see Methods). **C** Representative trajectory-dependent platelet [Ca^2+^]_c_ profiles for trajectories T1, T4, and T14 (**B**). **D** CFD-derived velocity profiles for T1, T4, and T14. **E** CFD-derived strain rate profiles for T1, T4, and T14. **F** CFD-derived shear stress profiles for T1, T4, and T14. **G** Coordinate system showing trajectories T1, T4, and T14 and 10 μm Ø subsampling regions of interest as a function of position within the microfluidic. **H** Ca^2+^ sampling along T1, T4, and T14 for 10 μm Ø ROIs defined as Baseline—− 3 mm upstream of stenosis apex center-point; Acc1—at start of flow acceleration zone; Acc2—mid-acceleration zone; Acc3—end of flow acceleration zone; Apex—center-point of stenosis apex; Vortex—average [Ca^2+^]_c_ at center of predicted vortex zone for T14 only; Post-apex—3 mm downstream of stenosis apex center-point (*N* = 20 independent experiments)

To investigate the impact of supraphysiological hemodynamics on platelet activation, we utilized a set of stenosis microfluidic geometries designed to subject blood/platelets to supraphysiological velocity gradients dependent on flow rate (Q) and streamwise trajectory. This microfluidic assay allowed for exposure of whole or reconstituted human blood to defined flow velocity gradients in the absence of platelet-surface adhesion or plasma protein interactions (Additional file 1: Fig. S1). By specifically controlling stenosis entry angle (*θ*_e_), we were able to modulate blood flow acceleration while maintaining peak velocity at the stenosis apex and maintaining constant downstream velocities. Initial CFD modelling of blood flow (see “Methods”) for a prototypical stenosis geometry with *θ*_e_ = 80°, designed to subject blood/platelet samples to a maximal velocity gradient of 0.01 to 1 m s^−1^ at *Q* = 200 μL/min (Fig. 1A), predicted a highly heterogenous strain rate field with manifest zones of low $\dot{\gamma}$ ≤100 s^−1^ and adjacent zones of elevated $\dot{\gamma}$ ≥100,000 s^−1^; proximal to the upstream face of stenosis entry (Fig. 1B & Additional file 1: Fig. S2A & B). CFD particle tracing (*Q* = 200 μL/min) predicted that platelets will experience distinct velocity and strain rate profiles determined by their spanwise position on release (Fig. 1B & Additional file 1: Fig. S2A). Platelet trajectories were qualitatively confirmed by time-averaged imaging of CytoTracker© Green-labelled human platelets reconstituted with RBC at a hematocrit of 40% perfused through the *θ*_e_ = 80° geometry at *Q* = 200 μL/min (Additional file 1: Fig. S2C).

Based on our CFD modelling data, we hypothesized that platelets would exhibit activation states dictated by their streamwise trajectory and correlated hemodynamic profile. To test this hypothesis, we conducted time-averaged confocal platelet Ca^2+^ imaging (see “Methods”) of reconstituted blood samples at *Q* = 200 μL/min. We interrogated this imaging data by mapping a subset of CFD predicted trajectories onto the imaging data (see “Methods”) and analyzed platelet [Ca^2+^]_c_ profiles as a function of predicted trajectory. Figure 1C shows platelet [Ca^2+^]_c_ flux profiles for sample trajectories designated T1, T4, and T14 (chosen as representative centerline, mid-span, and near wall trajectories (Fig. 1B)) and demonstrates that platelets tracking within 10 μm of the sidewall (T14) exhibit a significant [Ca^2+^]_c_ flux initiating at the start of flow acceleration, while platelets within trajectories approaching mid-span and centerline (T4 and T1) showed no change in [Ca^2+^]_c_ above baseline. Correlated interrogation of trajectory-specific hemodynamic profiles demonstrated that trajectories within 10 μm of the sidewall (T14) display an initial velocity (*U*), strain rate ($\dot{\gamma}$), and shear stress (*τ*) “inflection point,” typified by a marked reduction in all three parameters followed by a rapid upstroke (Fig. 1D–F). This hemodynamic “inflection point” occurred at the start of the acceleration phase and was congruent with initiation of platelet [Ca^2+^]_c_ flux (Fig. 1C–F). Mid-span and centerline trajectories (T4 and T1) did not show this hemodynamic behavior, with a relatively smooth transition in all hemodynamic parameters and no associated [Ca^2+^]_c_ flux (Fig. 1C–F). Significantly, platelets tracking 10 μm (T14) from the sidewall experience steadystate $\dot{\gamma}$ and *τ* prior to the acceleration phase ~20 to ~80-fold higher than mid-span and centerline trajectories, suggesting that platelet [Ca^2+^]_c_ flux is triggered by the perturbation of hemodynamics at flow acceleration rather than steadystate $\dot{\gamma}$ and *τ* magnitude per se.

To obtain a statistical overview of the acceleration phase Ca^2+^ signaling phenomenon, we subsampled designated regions of interest (ROI) (Fig. 1G) along T1, T4, and T14 for *N* = 20 independent donor platelet samples (Fig. 1H). ROI sampling supported our single-profile studies (Fig. 1C) and indicated that T14 platelet [Ca^2+^]_c_ flux was initiated at the start of the acceleration phase (Acc1), with a significant shift from baseline of ~100 to ~408 nM (Fig. 1H). Maximal [Ca^2+^]_c_ was consistently reached at the relative midpoint of the acceleration phase (Acc2), with [Ca^2+^]_c_ approaching ~587 nM (~6-fold increase over baseline) that was a function of input Q (Fig. 1H and Additional file 1: S2D). This midpoint [Ca^2+^]_c_ maxima decreased approaching the stenosis apex, with peak [Ca^2+^]_c_ at the end of the acceleration phase (Acc 3) approaching ~325 nM (Fig. 1H). Significantly, [Ca^2+^]_c_ at stenosis apex (where $\dot{\gamma}$ and *τ* reached maximum) was ~2.5-fold lower (~235 nM) than at mid-acceleration (Fig. 1H). [Ca^2+^]_c_ at the center of the downstream flow vortex was ~199 nM (Fig. 1H), with [Ca^2+^]_c_ returning to baseline +3 mm post-apex (Fig. 1H). Supporting our single trajectory profiling data, T1 and T4 showed no significant change in platelet [Ca^2+^]_c_ above baseline across their respective trajectories (Fig. 1H).

**Fig. 2:**
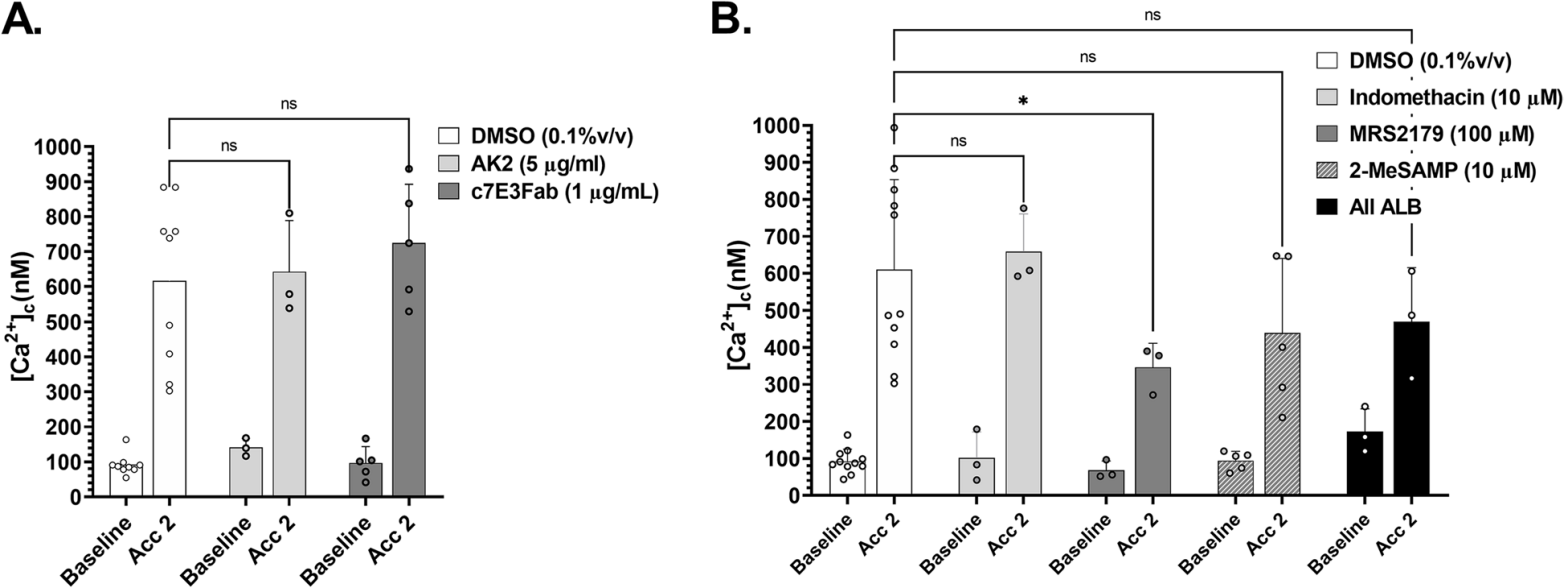
Trajectory-dependent [Ca^2+^]_c_ flux occurs independent of platelet adhesion receptor engagement and amplification loops. **A** Ca^2+^ sampling along T14 following treatment of reconstituted blood platelets with α-GPIb/V/IX blocking antibody AK2 (5 μg/mL) or α-α _IIb_β_3_ blocking antibody c7E3 Fab (Reopro©; 1 μg/mL). DMSO—vehicle control (*N* = 9 experiments); AK2—GPIb/V/IX blockade (*N* = 3 experiments); c7E3Fab (ReoPro)—integrin α_IIb_β_3_ blockade (*N* = 5 experiments). **B** Ca^2+^ sampling along T14 following treatment of reconstituted blood platelets with amplification loop inhibitors for 10 min. DMSO—vehicle control (*N* = 11 experiments); Indomethacin (10 μM) to inhibit COX1 (*N* = 3 experiments); MRS2179 [100 μM] to inhibit P2Y1 (*N* = 3 experiments); 2-MeSAMP [10 μM] to inhibit P2Y12 ADP dependent signaling (*N* = 5 experiments); ALB—all inhibitors combined (*N* = 3 independent experiments)

To eliminate a potential role for VWF binding interactions in mediating acceleration-dependent [Ca^2+^]_c_ flux, we conducted a series of experiments in which GPIb/V/IX to VWF binding was blocked with the blocking antibody AK2 (5 μg/mL) [Invitrogen] and platelet integrin α_IIb_β_3_ binding was blocked with c7E3Fab (Reopro©; 1 μg/mL). Neither AK2 nor c7E3Fab had any significant impact on acceleration-mediated platelet [Ca^2+^]_c_ flux (Fig. 2A). Similarly, blockade of canonical platelet amplification loops had minimal to no effect on platelet [Ca^2+^]_c_ flux under these conditions (Fig. 2B). Notably, platelet activation status as measured by integrin α_IIb_β_3_ activation (Pac1 binding) and P-selectin expression (AK-4), 6 mm post-stenosis apex, showed no significant elevation over baseline controls corroborating that acceleration-dependent platelet [Ca^2+^]_c_ flux was highly localized to the hemodynamic gradient and short range in effect (Additional file 1: Fig. S2E).

Collectively, these data demonstrate that platelets exhibit an acute mechanotransduction response to localized supraphysiological acceleration in free flow, independent of VWF and soluble agonist-mediated pathways. In addition, these data demonstrate that platelet activation is specifically dependent on platelet streamwise trajectory and correlated hemodynamic profile at acceleration start.

### Modification of acceleration profile directly modulates platelet [Ca^2+^]_c_ flux and aggregation dynamics

**Fig. 3:**
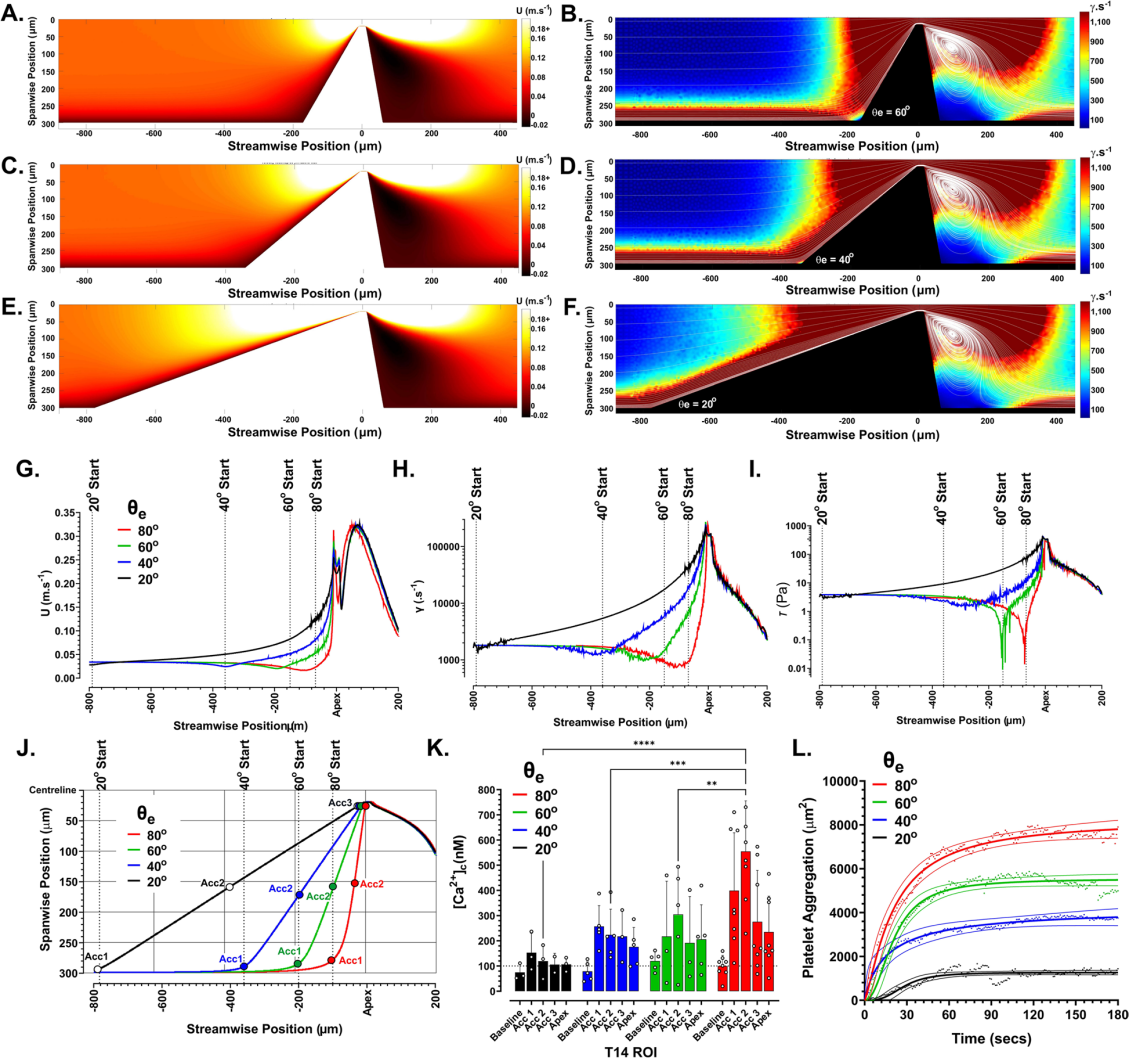
Constraint of platelet acceleration directly modulates [Ca^2+^]_c_ flux. **A**–**F** Color maps showing velocity (U m s^−1^) and strain rate ($\dot{\gamma}$ s^−1^) distributions within an *x*,*y* plane 50 μm from the microchannel floor (derived from CFD modelling of blood flow; *Q* = 200 μL/min) for *θ*_e_ = 60°, 40°, 20° (constant exit *θ* = 80°). White lines show predicted platelet trajectories (2 μm particles) (see “Methods”). **G** Predicted change in U for T14 as a function of *θ*_e_. **H** Predicted strain rate profiles for T14 as a function of *θ*_e_. **I** Predicted shear stress profiles for T14 as a function of *θ*_e_. **J** Coordinate system showing predicted change in trajectory T14 as a function of *θ*_e_ and 10 μm Ø subsampling regions of interest (ROI) as a function of position within the microfluidic. **K** Ca^2+^ sampling along T14 as a function of *θ*_e_ for 10 μm Ø ROIs defined as: Baseline—− 3 mm upstream of stenosis apex center-point; Acc1—at start of flow acceleration zone; Acc2—mid-acceleration zone; Acc3—end of flow acceleration zone (*θ*_e_ = 20° [*N* = 3 experiments]; *θ*_e_ = 40° [*N* = 5 experiments]; *θ*_e_ = 60° [*N* = 5 experiments]; *θ*_e_ = 80° [*N* = 8 experiments]). **L** Platelet aggregation at stenosis apex as a function of *θ*_e_. Curves shown are [Agonist] vs. response Variable slope (four parameters) least squares fit + 95% CI. Dots represent individual data sets from *N* = 5 independent experiments

To further explore the way in which acceleration profile impacts platelet [Ca^2+^]_c_ flux, we fabricated an additional series of symmetrical stenosis geometries with *θ*_e_ varying from *θ*_e_ = 20°, 40°, and 60° in order to progressively limit near wall (T14) acceleration in isolation from apex and downstream hemodynamics (Fig. 3A–F). CFD modelling of these geometries confirmed that *θ*_e_-dependent changes in the $\dot{\gamma}$ field were isolated from downstream effects, such that $\dot{\gamma}$ was equivalent at and downstream of the stenosis apex (Fig. 3A–F). CFD particle tracing also verified no significant change in apex-to-downstream platelet trajectories or vortex formation as a function of *θ*_e_ (Fig. 3B, D & F). Changes in all hemodynamic parameters were restricted to the acceleration phase of flow, with reduction in *θ*_e_ significantly modifying the velocity gradient such that platelets tracking T14 were predicted to undergo a reduction in peak acceleration and $\dot{\gamma}$-gradient (Additional file 1: Fig. S3A & B); with more gradual velocity and $\dot{\gamma}$ profiles (Fig. 3G, H). The *θ*_e_ = 60° geometry exhibited a *τ* “inflection point” equivalent to that seen for *θ*_e_ = 80° (Fig. 3I). Significantly, transitioning from *θ*_e_ = 60° to 40° resulted in a complete loss of the *τ* “inflection point,” with an order of magnitude increase in applied *τ* proximal to acceleration start (Fig. 3I). In correlation with the observed shift in *τ* profile as function of *θ*_e_, platelet [Ca^2+^]_c_ flux across the acceleration phase was reduced for *θ*_e_ = 20° and 40°, with θ_e_ = 20° showing no significant [Ca^2+^]_c_ flux over baseline controls (Fig. 3J, K). Overall, [Ca^2+^]_c_ profiles increased directly with increasing *θ*_e_ (Fig. 3K). Notably, there was no significant difference in [Ca^2+^]_c_ maxima for *θ*_e_ = 60° and 80°, suggesting that the observed hemodynamic “inflection point” is a key driver of the signaling response (Fig. 3K).

To explore the downstream functional effects of [Ca^2+^]_c_ flux as a function of acceleration profile, we developed a modified platelet aggregation assay (see “Methods”) in which the downstream face of the stenosis geometries were focally derivitized with purified human VWF (10 μg/mL), while all of the upstream components including the flow acceleration zone were blocked with 10%w/v bovine serum albumin (BSA) to prevent surface adhesion (Additional file 1: Fig. S1C & D). This approach allowed for the acceleration-dependent platelet [Ca^2+^]_c_ flux phase to be dissociated from possible GPIb/V/IX receptor engagement (Additional file 1: Fig. S1C & D). Significantly, using this approach, we demonstrate that downstream VWF-dependent platelet aggregation rate and extent directly correlate with acceleration profile (*θ*_e_) in free flow, demonstrating that platelet mechanotransduction of flow acceleration has a direct and localized effect on platelet aggregation (Fig. 3L & Additional file 1: S3C).

### Extensional strain is the primary trigger of platelet [Ca^2+^]_c_ flux in the absence of adhesion receptor engagement

**Fig. 4:**
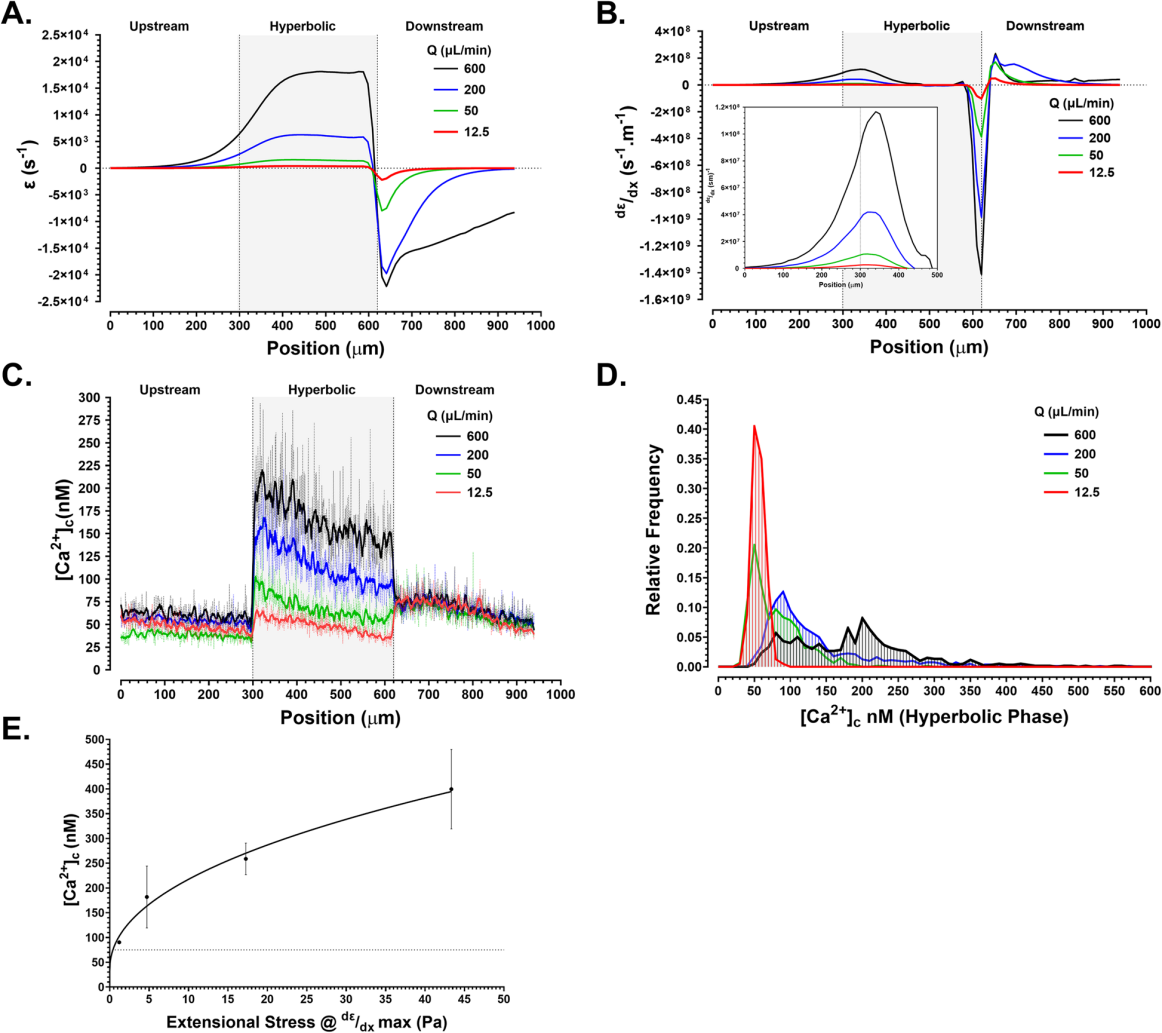
Threshold extensional strain triggers platelet [Ca^2+^]_c_ flux independent of wall shear stress. **A** CFD-derived extensional strain rate profile at hyperbolic centerline for *Q* = 12.5, 50, 200, and 600 μL/min. Grey region defines the hyperbolic phase. **B** CFD-derived rate of change in extensional strain rate profile at hyperbolic centerline. Inset—zoomed view of hyperbolic entry. Grey region defines the hyperbolic phase. **C** Human platelet [Ca^2+^]_c_ as a function of *Q*. Dotted line represents baseline [Ca^2+^]_c_ cutoff = 75 nM. Solid lines are a rolling average (2nd-order smoothing—10 neighbors). Grey region defines the hyperbolic phase. Note that Ca^2+^ activation is on/off such that it initiates at the beginning of the Hyperbolic zone (constant $\dot{\varepsilon}$) and immediately “shuts off” upon ejection into the Downstream expansion zone ($\dot{\varepsilon}$ = 0). **D** Frequency histogram showing the distribution of platelet [Ca^2+^]_c_ (10 nM bins) within the first 100 μm of Hyperbolic flow as a function of applied $\dot{\varepsilon}$. *Q* = 12.5 μL/min (*N* = 3 experiments); *Q* = 50 μL/min (*N* = 3 experiments); *Q* = 200mL/min (*N* = 4 experiments); *Q* = 600 μL/min (*N* = 4 experiments). Dotted line represents baseline [Ca^2+^]_c_ cutoff = 75 nM. **E** Platelet [Ca^2+^]_c_ as a function of applied extensional stress (Pa) at maximum $d\varepsilon \left/ d x\right.$ for *Q* = 12.5, 50, 200, and 600 μL/min. Dotted line represents baseline [Ca^2+^]_c_ cutoff = 75 nM. [Agonist] vs. response Variable slope (four parameters) Least squares fit + 95% CI shown of *N* = 4 independent experiments. Note that maximal [Ca^2+^]_c_ at *σ* = 43.3 Pa ranges between ~220 and ~600 nM.

Having demonstrated that platelets in free flow (in the absence of adhesion receptor engagement) undergo significant and rapid [Ca^2+^]_c_ flux in direct response to free-flow acceleration, we hypothesized that the extensional strain rate component of flow, through direct deformational effects on platelet structure, may be the key driver of this mechanotransduction mechanism. To test this hypothesis, we developed a hyperbolic microfluidic assay (see “Methods”) that subjects isolated human platelets to quasi-homogenous extensional strain rate (Cauchy strain rate, $\dot{\varepsilon}$) as a function of input flow rate (*Q*) (Additional file 1: Fig. S4). The hyperbolic geometry was developed to generate peak $\dot{\varepsilon}$ (based on analytical solution) ranging between 318 s^−1^ (*Q* = 12.5 μL/min) to >16,013 s^−1^ (*Q* = 600 μL/min) closely approximating the overall extensional strain component experienced in the stepped microfluidic geometries (Additional file 1: Fig. S2F). This approach allowed us to investigate the impact of $\dot{\varepsilon}$ in isolation from shear strain. Isolated human platelets resuspended in modified Tyrodes + CaCl_2_ (1 mM) buffer containing 0.5%w/v methylcellulose (*v* = 0.004±0.015 Pa.s; *ρ* = 1016 kgm^−3^) were perfused through this device at *Q* = 12.5, 50, 200, and 600 μL/min. CFD modelling of centerline flow (corresponding to the platelet [Ca^2+^]_c_ sampling region) through the hyperbolic microfluidic demonstrated that $\dot{\varepsilon}$ plateaued within 100 μm of the hyperbolic throat at 396 s^−1^, 1582 s^−1^, 6243 s^−1^, and 18,118 s^−1^ for *Q* = 12.5, 50, 200, and 600 μL/min respectively (Fig. 4A). Platelets displayed a step-wise increase in [Ca^2+^]_c_ flux as a function of *Q* and applied $\dot{\varepsilon}$, with a significant right shift in the overall platelet [Ca^2+^]_c_ frequency distribution (Fig. 4C, D). Peak platelet [Ca^2+^]_c_ directly correlated with the maximal rate of change of $\dot{\varepsilon}$ ($d\varepsilon \left/ d x\right.$) (Fig. 4B, C). [Ca^2+^]_c_ flux was triggered at applied extensional stresses >1.2 Pa, with maximal [Ca^2+^]_c_ flux ranging between 314 and 560 nM at an applied extensional stress of 43.3Pa ($d\varepsilon \left/ d x \right.$ = 1.1 × 10^8^ s^−1^ m^−1^); approaching that seen with the *θ*_e_ = 80° acceleration geometry (Fig. 4E). Significantly, $\dot{\varepsilon}$-driven [Ca^2+^]_c_ flux exhibited rapid On/Off behavior, such that ejection of the platelet sample into the downstream non-hyperbolic expansion resulted in immediate cessation of [Ca^2+^]_c_ flux at $d\varepsilon \left/ d x\right.$ ≤ −3.0 × 10^8^ s^−1^ m^−1^ (Fig. 4A–C). Notably, platelet [Ca^2+^]_c_ within the hyperbolic zone exhibited a decline in magnitude over time of ~50% that correlated with $d\varepsilon \left/ d x\right.$ approaching 0 s^−1^ m^−1^ (steadystate $\dot{\varepsilon}$) (Fig. 4B, C), suggesting that this mechanotransduction process is rapidly downregulated once steadystate $\dot{\varepsilon}$ is achieved.

This data supports our hypothesis that $\dot{\varepsilon}$ is a key hemodynamic driver of free-flow platelet Ca^2+^ flux and demonstrates that $\dot{\varepsilon}$ can trigger maximal platelet [Ca^2+^]_c_ approaching 600 nM, in isolation from shear strain. We therefore define this platelet mechanotransduction phenomenon as “*E*xtensional *S*train *S*ensing ($\dot{\varepsilon}$-*S*).”

### $\dot{\varepsilon}$-S is dependent on mechanically gated calcium influx

**Fig. 5:**
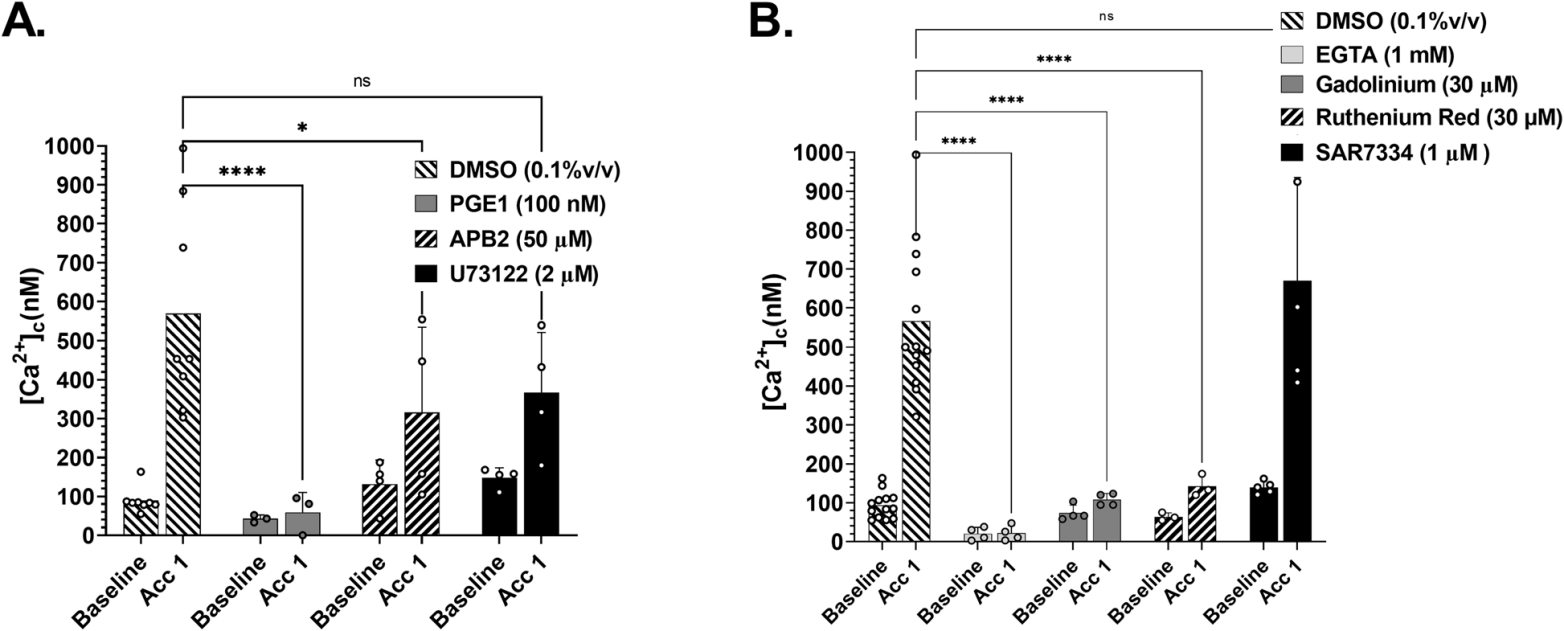
$\dot{\varepsilon}$-*S* [Ca^2+^]_c_ flux is dependent on mechanically gated calcium influx. **A** Ca^2+^ sampling along T14 in *θ* = 80° stenosis geometry following treatment of reconstituted blood (10 min) with DMSO − vehicle control (*N* = 8 experiments); PGE1 (100 nM) + Thephylline (10 mM) [*N* = 3 experiments]; APB2 (50 mM) to inhibit IP_3_ receptor (*N* = 4 experiments); U73122 (2 mM) to inhibit phospholipase C (*N* = 4 experiments). **B** Ca^2+^ sampling along T14 in *θ* = 80° stenosis geometry following treatment of reconstituted blood (10 min) with, DMSO − vehicle control (*N* = 13 experiments); EGTA (1 mM) to chelate extracellular Ca^2+^ (*N* = 4 experiments); Gadolinium (30 μM) [*N* = 4 experiments]; Ruthenium Red (30 μM) [*N* = 3 experiments]; SAR7334 (1 μM) to inhibit TRPC6 channels (*N* = 5 experiments)

Having demonstrated a role for flow acceleration and $\dot{\varepsilon}$ in triggering platelet [Ca^2+^]_c_ flux, we next explored the possible mechanism(s) of $\dot{\varepsilon}$-*S* signal transduction. Initial experiments in the *θ*_e_ = 80° (Q = 200 μL/min) geometry in which platelet activation was inhibited with PGE_1_ (100 nM) + Theophylline (10 mM) demonstrated that $\dot{\varepsilon}$-*S* [Ca^2+^]_c_ flux is an active signal transduction process and is regulated by intracellular [cAMP] (Fig. 5A). To investigate whether Ca^2+^ signaling was dependent on canonical IP_3_-mediated store release, we treated reconstituted blood samples with U73122 (2 μM) to inhibit IP_3_ generation by phospholipase C (PLC), or with the IP_3_ receptor blocker 2-Aminoethoxydiphenyl borate (APB2; 50 μM) to inhibit store release. Figure 5A demonstrates that U73122 had no significant effect on $\dot{\varepsilon}$-*S* [Ca^2+^]_c_ flux, while APB2 had a minimal effect, suggesting that IP_3_-mediated Ca^2+^ store release is not the major driver of $\dot{\varepsilon}$-*S* signal transduction. We therefore hypothesized that $\dot{\varepsilon}$-*S* [Ca^2+^]_c_ flux is primarily driven by extracellular Ca^2+^ influx. To explore this, platelets + RBC were reconstituted in modified Tyrodes buffer in the presence of EGTA/Mg^2+^ (1 mM). Figure 5B demonstrates that chelation of extracellular Ca^2+^ completely suppressed baseline and $\dot{\varepsilon}$-*S* [Ca^2+^]_c_, with [Ca^2+^]_c_ = ~22 nM; a ~78% reduction compared to baseline controls. Given this dependency on extracellular Ca^2+^ influx, we posited that cationic mechanosensitive channels (MSCs) may be the primary driver(s) of $\dot{\varepsilon}$-*S* [Ca^2+^]_c_. Figure 5B demonstrates that reconstituted blood samples treated with Gadolinium (30 μM) or Ruthenium Red (30 μM), to non-specifically block MSC-mediated Ca^2+^ influx, almost completely inhibited $\dot{\varepsilon}$-*S* [Ca^2+^]_c_ flux in response to flow acceleration (*θ*_e_ = 80°). Inhibition of the SOCE-independent nonselective cation entry channel TRPC6 [19] with the selective antagonist SAR7334 (1 μM) had no significant effect on $\dot{\varepsilon}$-*S* [Ca^2+^]_c_ (Fig. 5B). Taken together, these data suggest that $\dot{\varepsilon}$-*S* signal transduction is mediated by an MSC-mediated process, independent of SOCE and TRPC6.

### $\dot{\varepsilon}$-S is dependent on the mechanosensitive channel Piezo1

**Fig. 6:**
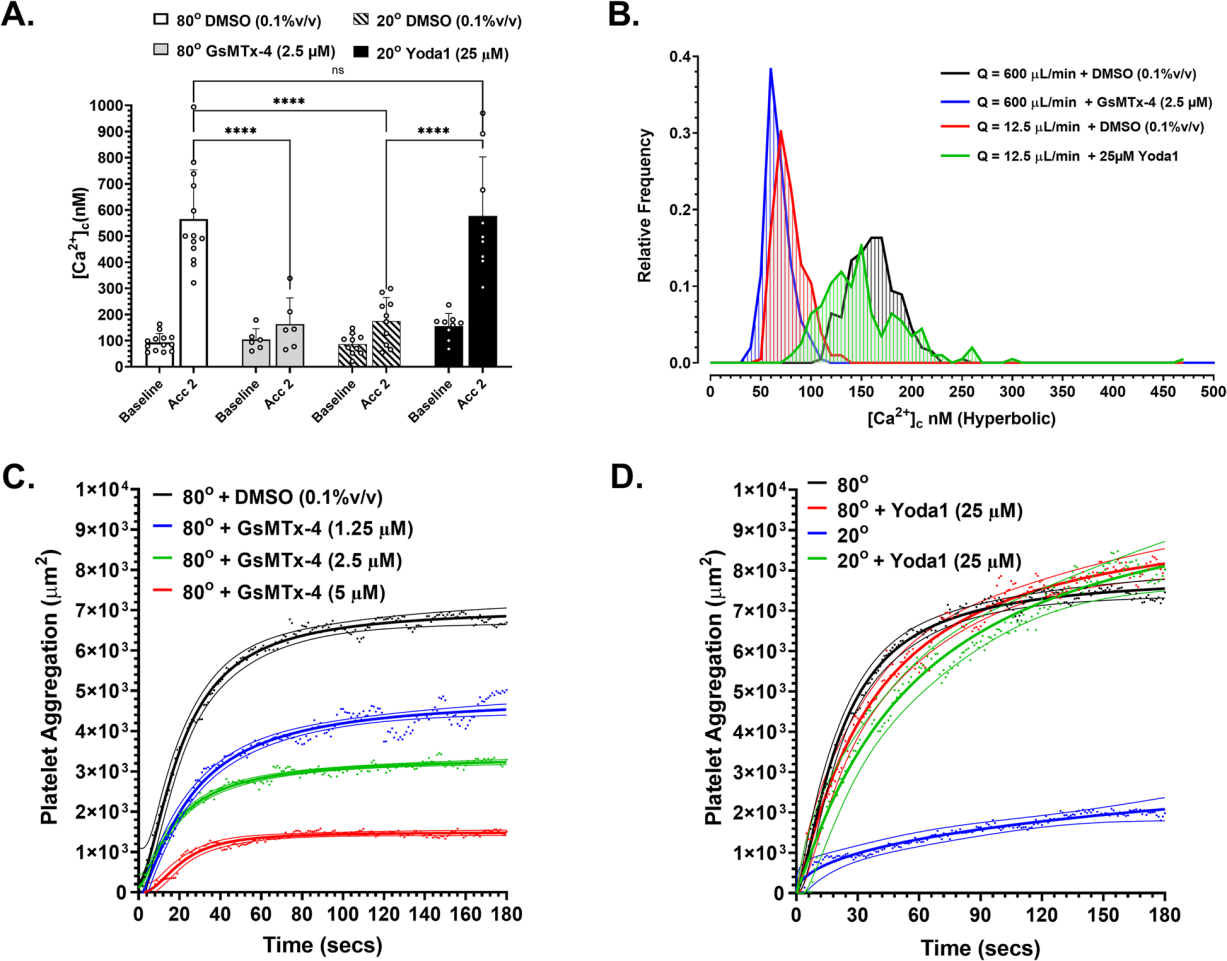
$\dot{\varepsilon}$-*S* [Ca^2+^]_c_ flux is dependent on the mechanically gated calcium channel Piezo1. **A** Ca^2+^ sampling along T14 in *θ* = 80° stenosis geometry following treatment of reconstituted blood (10 min) with, DMSO − vehicle control (*N* = 13 experiments); GsMTX-4 (2.5 μM) [*N* = 6 experiments] and Ca^2+^ sampling along T14 in *θ* = 20° stenosis geometry following treatment of reconstituted blood (10 min) with, DMSO (*N* = 10 experiments); Yoda1 (25 μM) [*N* = 9 experiments]. **B** Frequency histogram showing the distribution of platelet [Ca^2+^]_c_ (10 nM bins) within Hyperbolic flow at *Q* = 600 μL/min: DMSO (0.1%v/v); and GsMTx-4 (2.5 μM) and *Q* = 12.5 μL/min DMSO (0.1%v/v); and Yoda1 (25 μM) [*N* = 3 experiments]. **C** Platelet aggregation at stenosis apex *θ*_e_ = 80° following treatment of DiOC_6_ labelled human whole blood with, DMSO (*N* = 4 experiments); GsMTX-4 (1.25 μM) [*N* = 4 experiments]; GsMTX-4 (2.5 μM) [*N* = 4 experiments]; GsMTX-4 5 μM [*N* = 4 experiments]. Curves shown are [Agonist] vs. response Variable slope (four parameters) least squares fit + 95% CI. **D** Platelet aggregation at stenosis apex *θ* = 20° and 80° following treatment of DiOC_6_-labelled human whole blood with DMSO (*N* = 4 experiments); Yoda1 (25 μM) [*N* = 3 experiments]. Curves shown are [Agonist] vs. response Variable slope (four parameters) least squares fit + 95% CI

The platelet-expressed MSC, Piezo1, as a direct force sensor [20], has been implicated in shear-mediated platelet thrombus formation [21]. We therefore postulated that Piezo1 was acting as the primary MSC driving $\dot{\varepsilon}$-*S* [Ca^2+^]_c_ flux and as the direct $\dot{\varepsilon}$ sensor at free-flow acceleration. To investigate this, we conducted both stenosis-dependent and hyperbolic flow experiments in which reconstituted blood and isolated platelets respectively, were treated with the spider venom peptide *Grammostola spatulata* mechanotoxin 4 (GsMTx*-*4; Invitro Technologies); a non-specific MSC antagonist widely used to explore Piezo1 function [22]. Treatment of reconstituted blood with GsMTx-4 (2.5 μM) 10 min prior to perfusion through the *θ*_e_ = 80° geometry (*Q* = 200 μL/min) significantly (*P* < 0.0001) inhibited $\dot{\varepsilon}$-*S* [Ca^2+^]_c_ flux, with a ~88% reduction in [Ca^2+^]_c_ compared to control (Fig. 6A). In addition, $\dot{\varepsilon}$-*S* [Ca^2+^]_c_ flux at $\dot{\varepsilon}$ = 16,013 s^−1^ (hyperbolic flow) was completely inhibited by 2.5 μM GsMTx-4 (Fig. 6B).

Given the lack of specificity of GsMTx-4 and to more definitively define a role for Piezo1 in mediating $\dot{\varepsilon}$-*S* [Ca^2+^]_c_ flux, we examined the capacity for the Piezo1-specific agonist Yoda1 (Sigma Aldrich) [23] to amplify $\dot{\varepsilon}$-*S* signaling under subthreshold flow acceleration (*θ*_e_ = 20°) and subthreshold $\dot{\varepsilon}$ = 197 s^−1^. Treatment of reconstituted blood with Yoda1 (25 μM) at subthreshold acceleration resulted in a return of $\dot{\varepsilon}$-*S* [Ca^2+^]_c_ flux to that observed for *θ*_e_ = 80° controls, with [Ca^2+^]_c_ ≥600 nM (Fig. 6A). In parallel, treatment of isolated platelets with Yoda1 (25 μM) at a subthreshold $\dot{\varepsilon}$ = 318 s^−1^ resulted in a return of $\dot{\varepsilon}$-*S* [Ca^2+^]_c_ flux (~79% increase) approaching that observed for$\dot{\ \varepsilon }$ = 16,013 s^−1^ controls (Fig. 6B).

Finally, we explored the role of Piezo1 in mediating $\dot{\varepsilon}$-*S* [Ca^2+^]_c_ flux-affected platelet aggregation in response to flow acceleration. Figure 6C demonstrates that GsMTx-4 can inhibit free-flow acceleration-mediated platelet aggregation in a concentration-dependent manner with 2.5 μM GsMTx-4 resulting in a ~47% reduction and 5 μM GsMTx-4 a ~76% reduction in endpoint aggregation at threshold acceleration (*θ*_e_ =80°). Conversely, treatment of whole blood with Yoda1 (25 μM) under subthreshold acceleration (*θ*_e_ = 20°) led to an ~8-fold increase in focal platelet aggregation, matching both aggregation rate and maxima of *θ*_e_ = 80° controls (Fig. 6D). Treatment of whole blood with Yoda1 (25 μM) under maximal acceleration (*θ*_e_ = 80°) led to no further increase over controls (Fig. 6D).

Taken together, these data demonstrate that Piezo1-mediated Ca^2+^ influx is a critical component of $\dot{\varepsilon}$-*S* signal transduction and suggest that platelet Piezo1 is the primary force sensor facilitating adhesion-independent platelet Ca^2+^ activation in response to both blood flow acceleration and $\dot{\varepsilon}$.

### $\dot{\varepsilon}$-S is critically dependent on concomitant ATP gated P2X1 Ca^2+^ influx

**Fig. 7:**
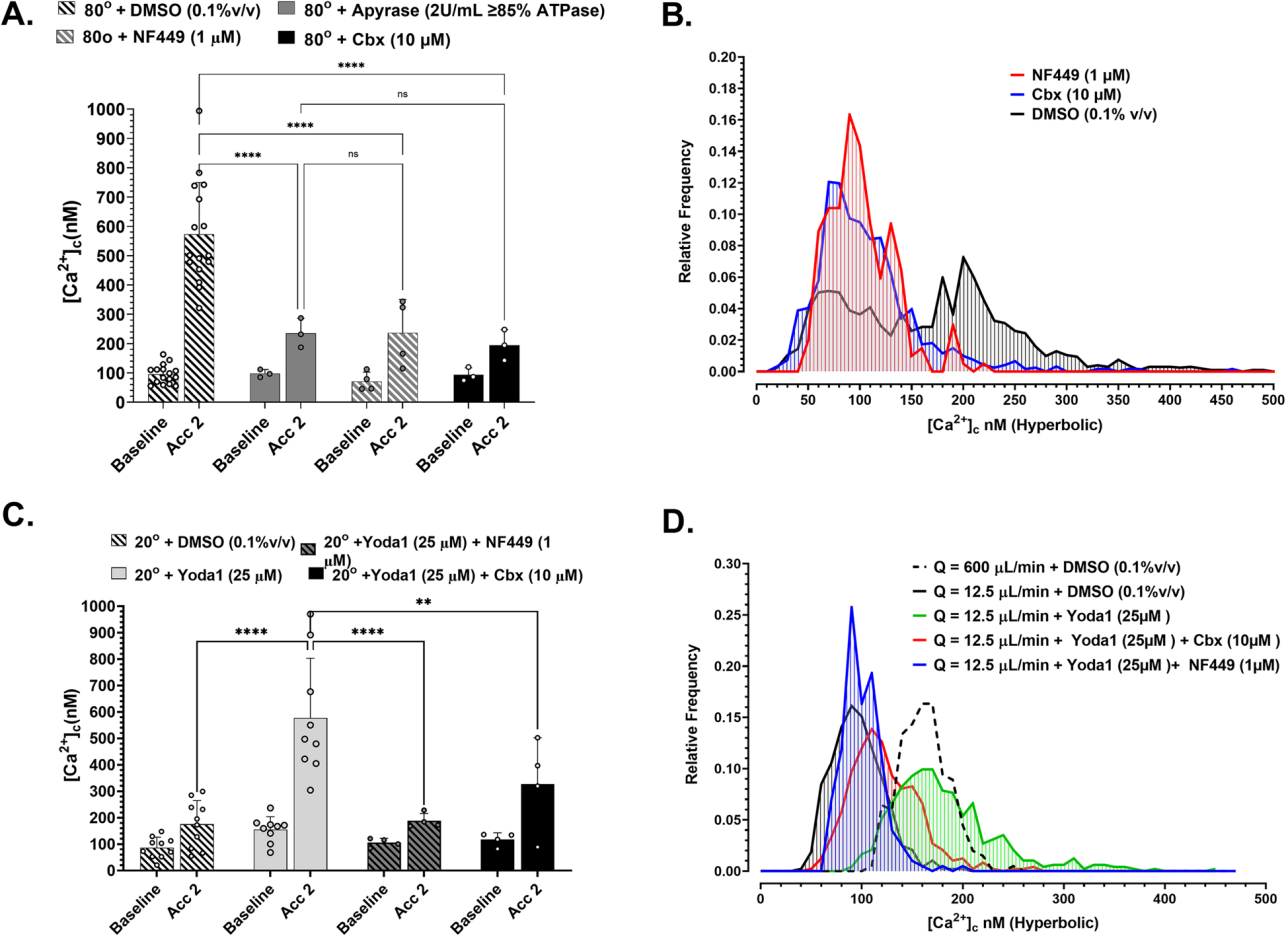
$\dot{\varepsilon}$-*S* [Ca^2+^]_c_ flux is dependent on Panx1 and P2X1. **A** Ca^2+^ sampling along T14 in *θ* = 80° stenosis geometry following treatment of reconstituted blood (10 min) with DMSO (*N* = 16 experiments); Apyrase (2 U/mL ≥ 85% ATPase activity) [*N* = 3 experiments]; NF449 (1 μM) [*N* = 4 experiments]; Carbenoxolone (10 μM) [*N* = 3 experiments]. **B** Frequency histogram showing the distribution of platelet [Ca^2+^]_c_ (10 nM bins) within Hyperbolic flow at *Q* = 600 μL/min: DMSO (0.1%v/v); NF449 (1 μM); Carbenoxolone (Cbx; 10 μM) [*N* = 3 independent experiments]. **C** Ca^2+^ sampling along T14 in *θ* = 20° stenosis geometry following treatment of reconstituted blood (10 min) with DMSO (*N* = 10 experiments); Yoda1 (25 μM) [*N* = 9 experiments]; Yoda1 (25 μM) + Cbx (10 μM) [*N* = 3 experiments]; Yoda1 (25 μM) + NF449 (1 μM) [*N* = 4 experiments]. **D** Frequency histogram showing the distribution of platelet [Ca^2+^]_c_ (10 nM bins) within Hyperbolic flow at *Q* = 600 μL/min

Several studies have demonstrated a role for mechanical and chemical activation of Piezo1 in driving autocrine ATP release and subsequent purinergic receptor activation [24, 25]. In the case of platelets, autocrine release of ATP and activation of the fast ATP-gated P2X1 cation channel has been demonstrated to be important for shear-mediated platelet activation and thrombus formation [26]. We therefore hypothesized that Piezo1 acts as the direct $\dot{\varepsilon}$ sensor and couples to P2X1 activation through local ATP release amplifying $\dot{\varepsilon}$-*S* [Ca^2+^]_c_ flux. To test this hypothesis, we assessed the effect of high [apyrase] (2 U/mL ≥85% ATPase; Sigma Aldrich) and direct inhibition of P2X1 with the highly specific antagonist NF449 (1 μM) [Merck] on flow acceleration (*θ*_e_ = 80°)-dependent $\dot{\varepsilon}$-*S* [Ca^2+^]_c_ flux. Figure 7A demonstrates that apyrase and NF449 led to a ~71% and ~66% reduction, respectively in $\dot{\varepsilon}$-*S* [Ca^2+^]_c_ flux. By extrapolation, in the absence of ATP/P2X1-dependent amplification, Piezo1 alone contributes ~29 to 35% (Δ[Ca^2+^]_c_ = 101–138 nM) of total $\dot{\varepsilon}$-*S* [Ca^2+^]_c_ flux under the conditions tested. Platelet aggregation at flow acceleration (*θ*_e_ = 80°) showed a ~30% reduction in endpoint aggregate size in the presence of NF449 (1 μM) (Fig. S5). Hyperbolic flow experiments demonstrated that at $\dot{\varepsilon}$ = 16,013 s^−1^, $\dot{\varepsilon}$-*S* [Ca^2+^]_c_ flux was almost completely inhibited by NF449 (1 μM) (Fig. 7B).

Based on these data and published data describing a role for the ATP permeable hemichannel pannexin1 (Panx1) in mediating platelet ATP release, Ca^2+^ influx, platelet aggregation, and thrombus formation [27], we examined the effect of the Panx1 inhibitor carbenoxalone (Cbx; 10 μM) in modulating $\dot{\varepsilon}$-*S* [Ca^2+^]_c_ flux. Figure 7A and B demonstrate that Cbx (10 μM) lead to an ~80% reduction in $\dot{\varepsilon}$-*S* [Ca^2+^]_c_ flux equivalent to that observed for apyrase and NF449 treatment. Cbx (10 μM) also inhibited platelet aggregation at (*θ*_e_ = 80°) flow acceleration and was typified by observable cyclic embolization indicative of unstable platelet aggregation (Additional file 1: Fig. S5).

Finally, to explore the interaction between Piezo1 gating and ATP-P2X1 mediated $\dot{\varepsilon}$-*S* [Ca^2+^]_c_ flux, we conducted a series of combinatorial experiments under subthreshold flow acceleration (*θ*_e_ = 20°) and subthreshold $\dot{\varepsilon}$ = 318 s^−1^ in which reconstituted blood or isolated platelets respectively were treated with Yoda1 (25 μM) to potentiate Piezo1 gating in combination with NF449 (1 μM) or Cbx (10 μM). Figure 7C and D demonstrate that under these subthreshold conditions Yoda1 (25 μM) mediated restoration of $\dot{\varepsilon}$-*S* [Ca^2+^]_c_ flux was inhibited by ~80% in the presence of NF449 (1 μM) and ~51% by Cbx (10 μM). Taken together, these data demonstrate that $\dot{\varepsilon}$-*S* [Ca^2+^]_c_ flux is mediated by mechanical activation of Piezo1 in response to $\dot{\varepsilon}$ leading to downstream gating of Panx1 and localized release of ATP, triggering subsequent P2X1 gating and Ca^2+^ influx. Overall, these data suggest that P2X1 plays a significant role in amplifying [Ca^2+^]_c_ influx initially triggered by Piezo1 mechanosensing.

### Type II PI3 kinase C2α activity critically modulates $\dot{\varepsilon}$-S

**Fig. 8:**
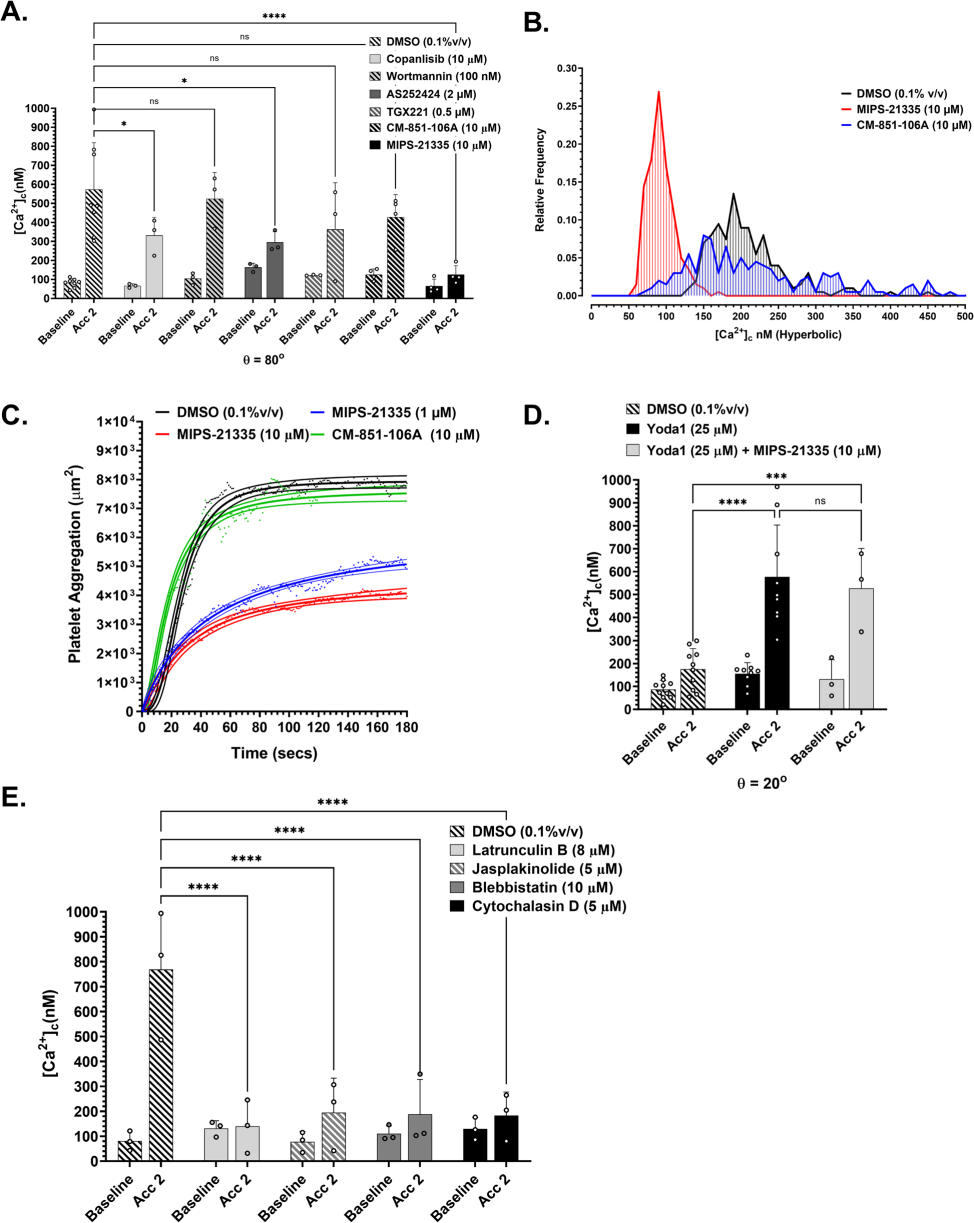
Type II PI3 kinase C2 α activity modulates $\dot{\varepsilon}$-*S* [Ca^2+^]_c_ flux. **A** Ca^2+^ sampling along T14 in *θ* = 80° stenosis geometry following treatment of reconstituted blood (10 min) with DMSO (0.1%v/v) [*N* = 8 experiments]; Copanlisib (10 μM) [*N* = 3 experiments]; Wortmannin (100 nM) [*N* = 3 experiments]; AS252424 (2 μM) [*N* = 3 experiments]; TGX221 (0.5 μM) [*N* = 3 experiments]; MIPS-21335 (10 μM) [*N* = 4 experiments]; CM-851-106A (10 μM) [*N* = 4 experiments]. **B** Frequency histogram showing the overall distribution of platelet [Ca^2+^]_c_ (10 nM bins) within Hyperbolic flow at *Q* = 600 μL/min: DMSO (0.1%v/v); MIPS-21335 (10 μM); CM-851-106A (10 μM). **C** Platelet aggregation at stenosis apex *θ* = 80° following treatment of DiOC_6_ labelled human whole blood with, DMSO (0.1%v/v) [*N* = 6 experiments]; MIPS-21335 (10 μM) [*N* = 6 experiments]; MIPS-21335 (1 μM) [*N* = 3 experiments]; CM-851-106A (10 μM) [*N* = 4 experiments]. Curves shown are [Agonist] vs. response Variable slope (four parameters) least squares fit + 95% CI. **D** Ca^2+^ sampling along T14 in *θ* = 20° stenosis geometry following treatment of reconstituted blood (10 min) with DMSO (0.1%v/v) [*N* = 10 experiments]; Yoda1 (25 μM) [*N* = 9 experiments]; Yoda1 (25 μ M) + MIPS-21335 (10 μM) [*N* = 3 experiments]. **E** Ca^2+^ sampling along T14 in *θ*_e_ = 80° stenosis geometry following treatment of reconstituted blood (10 min) with DMSO (0.1%v/v) [*N* = 3 experiments]; Latrunculin B (8 μM) [*N* = 3 experiments]; Jasplakinolide (5 μM) [*N* = 3 experiments]; Blebbistatin (10 μM) [*N* = 3 experiments]; Cytochalasin D (5 μM) [*N* = 3 experiments]

Using both inducible knockdowns in mice and first in class inhibitors of the type II PI3 kinase (PI3K) C2*α* isoform, we have previously demonstrated that velocity gradient-dependent platelet aggregation is significantly dependent on PI3KC2α activity [28, 29]. In addition, modelling studies have suggested a role for membrane lipid composition and in particular phosphoinositides in modulating Piezo1 conformation and gating [30]. We therefore hypothesized that PI3K isoforms and specifically PI3K C2α may be a key modulator of $\dot{\varepsilon}$-*S* signal transduction. To test this hypothesis, we conducted a broad screen of PI3K inhibitors and assessed their effect following perfusion of reconstituted blood through the *θ*_e_ = 80° geometry. Figure 8A demonstrates that neither Copanlisib (10 μM) [Sigma Aldrich] nor Wortmannin (100 nM) [Sigma Aldrich], as pan-specific PI3K inhibitors, showed significant inhibition of $\dot{\varepsilon}$-*S* [Ca^2+^]_c_ flux. Equally, neither the Type I PI3Kγ inhibitor AS252424 (2 μM) [Sigma Aldrich] nor the PI3Kβ inhibitor TGX221 (0.5 μM) [Sigma Aldrich] had significant effect on $\dot{\varepsilon}$-*S* [Ca^2+^]_c_ flux (Fig. 8A). In contrast, the PI3K C2α-specific inhibitor MIPS-21335 (10 μM) [29] completely inhibited $\dot{\varepsilon}$-*S* [Ca^2+^]_c_ flux with an ~88% reduction in [Ca^2+^]_c_, while its inactive analog CM-851-106A (10 μM) showed no inhibitory effect (Fig. 8A). Parallel investigation of the inhibitory effect of MIPS-21335 on isolated platelets as a function of $\dot{\varepsilon}$ = 16,013 s^−1^ supported this finding, demonstrating a significant role for PI3K C2α in modulating $\dot{\varepsilon}$-*S* [Ca^2+^]_c_ flux (Fig. 8B). In corroboration of our prior published studies [29], inhibition of PI3K C2α with MIPS-21335 significantly attenuated acceleration-dependent platelet aggregation with a ~50% reduction in endpoint aggregate size and an observable reduction in the initial rate of platelet aggregation at the *θ*_e_ = 80° stenosis geometry (Fig. 8C). In contrast, potentiation of Piezo1-mediated [Ca^2+^]_c_ influx with Yoda1 (25 μM) at subthreshold acceleration (*θ*_e_ = 20°) was not effected by MIPS-21335 (10 μM), suggesting that direct modulation of Piezo1 by Yoda1 uncouples the requirement for PI3K C2α activity and by extrapolation that PI3K C2α sits sequentially upstream of Piezo1 gating (Fig. 8D).

Finally, heterozygous inactivation of PI3K C2α in mice has been demonstrated to lead to defects in platelet thrombus formation with concomitant rigidification of the platelet plasma membrane and associated mislocation of several membrane cytoskeletal proteins [31]. We therefore posited that coupling of $\dot{\varepsilon}$ to $\dot{\varepsilon}$-*S* [Ca^2+^]_c_ flux is modulated by PI3K C2α-dependent cytoskeletal dynamics and associated membrane mechanics. As a preliminary test of this hypothesis, we ran a panel of pharmacological cytoskeletal inhibitors under conditions of threshold acceleration (*θ* = 80°). Figure 8E demonstrates that $\dot{\varepsilon}$-*S* [Ca^2+^]_c_ flux is critically dependent on active cytoskeletal dynamics such that inhibition of cytoskeletal polymerization (Cytochalasin D; 5 μM or Latrunculin B; 8 μM; Sigma Aldrich), disordering of polymeric actin (Jasplakinolide; 5 μM; Sigma Aldrich), or inhibition of myosin II activity (Blebbistatin; 10 μM; Sigma Aldrich) completely inhibited $\dot{\varepsilon}$-*S* signal transduction. Taken together, these data demonstrate that PI3K C2α plays a critical role in regulating $\dot{\varepsilon}$-*S* and by extrapolation suggests that membrane PI(3)P and membrane/cytoskeletal mechanics play a significant modulatory role coupling $\dot{\varepsilon}$ to platelet Piezo1 mechanosensing in free flow.

## Discussion

**Fig. 9:**
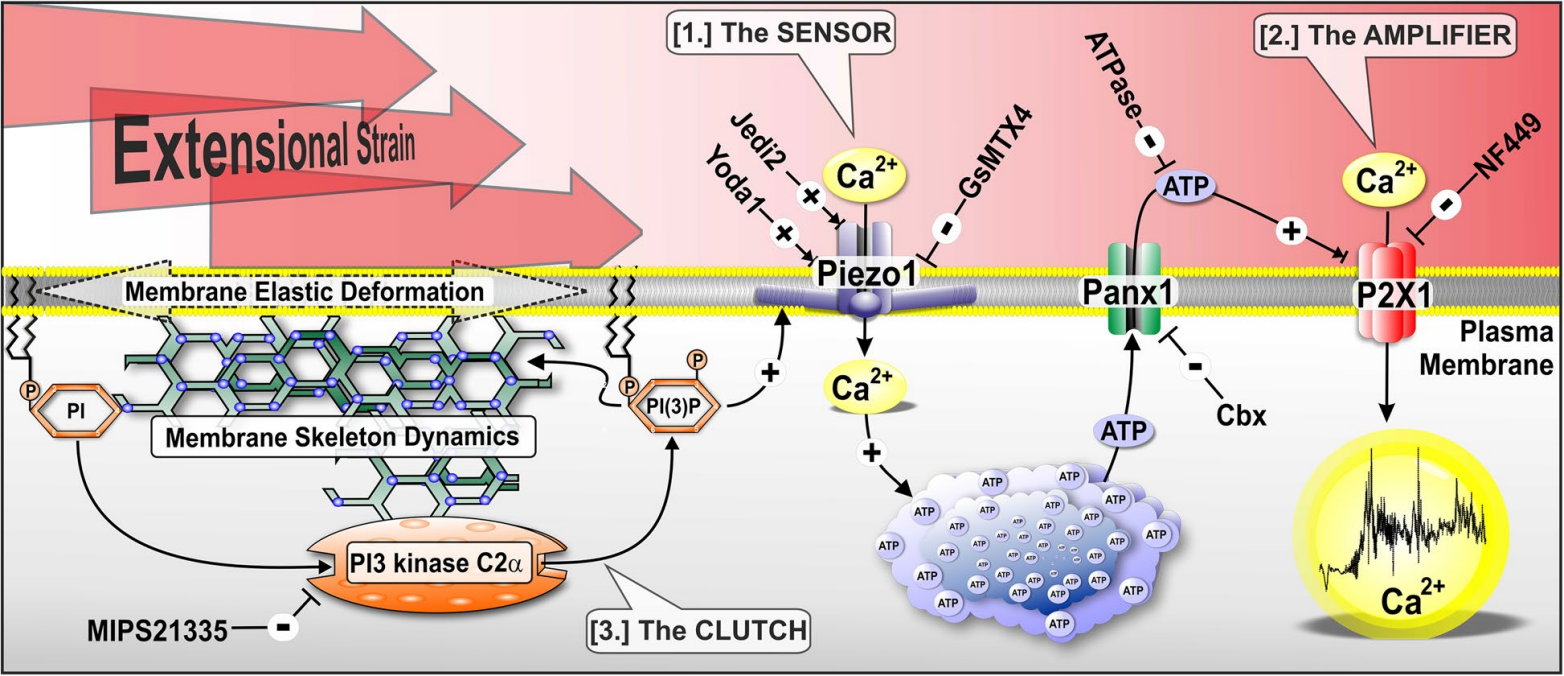
Working model of the *E*xtensional *S*train *S*ensing ($\dot{\varepsilon}$-*S*) Mechanosome. Extensional strain ($\dot{\varepsilon}$) generated by flow acceleration and acute changes in platelet trajectory impose a deformational load on the platelet plasma membrane and underlying spectrin-based membrane skeleton engaging the $\dot{\varepsilon}$-*S* mechanosome comprised of [1.] *The Sensor*—Piezo1 gating driven by deformational stress loading of the plasma membrane and membrane skeleton (membrane tension) leads to low level Ca^2+^ influx; [2.] *The Amplifier*—Piezo1-mediated Ca^2+^ influx triggers near-membrane ATP release via Pannexin1 (Panx1) triggering subsequent P2X1 activation, amplifying $\dot{\varepsilon}$-*S* [Ca^2+^]_c_ flux; [3.] *The Clutch*—A localized pool of PI(3)P, dependent on basal PI3-kinase C2α activity, modulates membrane skeleton composition, membrane elastic deformation, and associated force coupling through the plasma membrane to Piezo1

A large volume of work has explored the role of platelet adhesion receptors in mechanotransducing the external forces of blood flow, with wall shear rate/stress and compressive forces given specific attention [5, 6, 13]. These studies have led to an overall model of platelet activation under steadystate shear whereby initial platelet engagement with shear decrypted VWF, either free in plasma, expressed at the endothelial surface, or adhered to exposed extracellular collagen, leads to initial GPIb/V/IX-mediated mechanotransduction and subsequent integrin α_IIb_β_3_ outside-in signaling. Key to these mechanosensing processes are the effects of tensile and compressive forces on adhesion receptor conformation, and transmission of force to integral membrane proteins and the underlying cytoskeleton. While critical to overall platelet function and thrombus formation, this model does not consider the effects of blood flow hemodynamics on platelet activation and function in free flow. Here, we report for the first time (to our knowledge) that in the absence of canonical adhesion receptor engagement, VWF binding, and platelet amplification loops, platelets are capable of directly mechanosensing supraphysiological changes in blood flow acceleration and more specifically extensional strain rate ($\dot{\varepsilon}$). Significantly, we demonstrate that platelet activation occurs in response to $\dot{\varepsilon}$rather than peak shear stress magnitude. Using a pharmacological approach, we define an “*E*xtensional *S*train *S*ensing ($\dot{\varepsilon}$-*S*) mechanosome” comprised of three primary subunits: a principal mechanosensor in the form of membrane localized Piezo1, a coupled Ca^2+^ signal amplifier in the form of the fast ATP-gated cation channel P2X1, and a modulatory “clutch” comprised of the type II PI3 kinase C2α isoform (Fig. 9). Our findings have significant implications for our understanding of platelet function and thrombosis under pathological or device-induced supraphysiological hemodynamics, establishing that hemodynamic parameters in isolation, and specifically $\dot{\varepsilon}$, are sufficient to trigger platelet activation.

Regarding the $\dot{\varepsilon}$-*S* mechanosome, our pharmacological data support a key role for Piezo1 as the primary mechanosensor (Fig. 9) triggering $\dot{\varepsilon}$-*S* [Ca^2+^]_c_ flux under conditions of both free-flow trajectory-dependent acceleration and quasi-homogenous $\dot{\varepsilon}$. This finding is underpinned by transcriptomic and proteomic data demonstrating Piezo1 expression in human platelets [32, 33] and findings that platelet Ca^2+^ entry stimulated by physiological $\dot{\gamma}$ in the range of 1003–3989 s^−1^, is sensitive to both GsMTx-4 inhibition and Yoda1 potentiation [21]. Our data demonstrate that inhibition of Piezo1 with GsMTx-4 leads to effective inhibition of $\dot{\varepsilon}$- *S* [Ca^2+^]_c_ flux and a correlated inhibition of downstream functional platelet aggregation. Significantly, under subthreshold flow acceleration and $\dot{\varepsilon}$ (at which $\dot{\varepsilon}$-*S* [Ca^2+^]_c_ flux and aggregation were minimal), the Piezo1 agonist Yoda1 potentiated [Ca^2+^]_c_ flux and associated aggregation comparable to high acceleration and $\dot{\varepsilon\ }$controls. These findings are supported by studies showing that Yoda1 predominantly affects Piezo1 sensitivity and inactivation kinetics in response to mechanical stimulation, whereby Yoda1 acts as a “molecular wedge” amplifying force-induced Piezo1 conformational changes; lowering the channel threshold for activation [24, 34]. While our findings support prior studies for a role of Piezo1 in platelet function, there are some key differences: (i) Ilkan et al. [21] describe a set of experimental conditions in which human platelets were first surface immobilized via an anti-PECAM1 antibody prior to $\dot{\gamma}$ exposure and thus were unable to differentiate between surface-derived tensile stresses and direct effects of applied $\dot{\gamma}$. Furthermore, their study could not exclude inputs from morphological changes and associated signaling induced by surface adhesion [21]. In contrast, our studies define a role for Piezo1 in the direct mechanosensing of applied $\dot{\varepsilon}$ in the complete absence of surface adhesion and shear strain; (ii) Ilkan et al. [21] also describe the effects of steadystate (4-min duration) physiological $\dot{\gamma}$ that trigger stochastic [Ca^2+^]_c_ transients exhibiting significant lagtimes of ~1 min post-shear initiation [21]. In contrast, our data demonstrate that under non-steadystate supraphysiological $\dot{\gamma}$ gradients and quasi-homogenous $\dot{\varepsilon}$ conditions that Piezo1-mediated [Ca^2+^]_c_ flux occurs immediately upon $\dot{\varepsilon}$ exposure. These prior studies in combination with our own suggest that free-flow mediated effects on platelet Piezo1 gating may be further modulated by adhesion-dependent signaling and that platelet Piezo1 may require significantly higher threshold $\dot{\gamma}$, in the absence of surface adhesions, to trigger Ca^2+^ influx.

Our data support a role for ATP release via the Panx1 hemichannel and associated P2X1-mediated Ca^2+^ influx in $\dot{\varepsilon}$-*S*. Significantly, our data suggest that in the absence of ATP-P2X1 signaling that Piezo1 alone contributes only ~29–35% of total Ca^2+^ influx, indicating that P2X1 plays a major role in amplifying $\dot{\varepsilon}$-*S* [Ca^2+^]_c_. We demonstrate that blockade of P2X1 activation with the specific antagonist NF449 reduced endpoint aggregation in our system by ~30%, while concentration-dependent GsMTx-4 inhibition led to a 47–76% reduction in aggregate size. This apparent dichotomy between the contributions of P2X1 and Piezo1 to Ca^2+^ flux magnitude versus aggregation dynamics may indicate that receptor-mediated outside-in and soluble agonist signaling following platelet recruitment to the developing aggregate reduce the requirement for P2X1 amplification and in turn modify the contribution by Piezo1. Furthermore, once surface or platelet-platelet adhesions have been established, the effects of local hemodynamics on deformational stress loading of the platelet plasma membrane and underlying membrane skeleton are likely to be substantially different, with both compressive and tensile loading of adhesion receptors and platelet structures contributing to ongoing platelet Ca^2+^ flux. These findings demonstrating co-dependency between Piezo1 and P2X1 signaling in $\dot{\varepsilon}$-*S* are supported by accumulating evidence for a role for ATP release and P2 receptor signaling in mediating Piezo1-dependent mechanoregulation in a number of cell types, including vascular endothelial cells, and red blood cells [24, 25, 35, 36]. Notably, these prior studies using both pharmacologic and siRNA suppression strategies have shown a key role for the Panx1 hemichannel as the primary mediator of Piezo1-triggered ATP release at threshold shear stresses >3 Pa [35, 36].

There is accumulating evidence showing that membrane lipids have both positive and negative modulatory effects on Piezo1 function, with these effects likely due to alterations in global membrane properties such as bending rigidity or stiffness of the local lipid bilayer [30, 37, 38]. Specifically, molecular dynamics simulations of Piezo1 in complex lipid bilayers have implicated a role for membrane phosphoinositide phosphates (PIPs) in modulating Piezo1 conformation and gating [30]. These studies predict that negative lipid species including PI(3)P share common binding sites proximal to the Piezo1 cation channel pore [30]. Our data, for the first time (to our knowledge), demonstrate that type II PI3K C2α activity and by corollary PI3K C2α-generated lipids are critical mediators of $\dot{\varepsilon}$-*S* and by extrapolation Piezo1-mediated mechanosensing. Our findings are underpinned by previously published data from our laboratories demonstrating that platelet aggregation under moderate $\dot{\gamma}$ conditions ($\dot{\gamma}$ = 1800–22,500 s^−1^) was suppressed by both pharmacologic inhibition and inducible depletion of PI3KC2α [28, 29]. Significantly, these prior studies demonstrated that pharmacologic inhibition with the PI3KC2α-specific inhibitor MIPS-21335 reduced endpoint platelet aggregation (with and without platelet amplification loop blockade) approaching ~55% [29], matching the inhibitory effect in the present study (~50% reduction) under significantly more acute $\dot{\gamma}$ conditions ($\dot{\gamma}$ = 100–100,000 s^−1^). Given that we show that upstream $\dot{\varepsilon}$-*S* [Ca^2+^]_c_ flux and correlated downstream platelet aggregation scale with acceleration magnitude and Q (Fig. 3H & S2C), the equivalency of MIPS-21335 inhibition at moderate [28, 29] and supraphysiological hemodynamic gradients (Fig. 1A) suggests that PI3KC2α activity is absolutely required for $\dot{\varepsilon}$-*S*.

We demonstrate that $\dot{\varepsilon}$-*S* signaling is not affected by the pan-specific PI3K inhibitor Wortmannin, the pan-class I inhibitor Copanlisib, nor the type I isoform-specific PI3K inhibitors AS2524424 and TGX221, directed against PI3Kγ and PI3Kβ respectively; supporting a specific role for the type II PI3KC2α isoform. The apparent lack of effect of Wortmannin may be attributed in part to its overall lack of specificity for PI3K C2α, with an approximate IC_50_ for PI3K C2α of 400 nM [39], four times the concentration used in the present study. Notably, we demonstrate that potentiation of Piezo1 with Yoda1 completely abrogated the effect of specific PI3K C2α inhibition, suggesting that reduction in the mechanical threshold for Piezo1 activation by Yoda1 eliminates the requirement for PI3K C2α generated lipid species. Given the “molecular wedge” hypothesis of Yoda1 action [23, 34] and findings that strong protein-lipid interactions are required to anchor the bilayer around the curved shape of Piezo1 [40], alterations in local membrane curvature by Yoda1 may limit the requirement for PI3K C2α-derived PIPs.

Whereas class I PI3Ks reside mainly in the cytoplasm until recruited to active signaling complexes, the class II PI3Ks are largely constitutively associated with membrane structures and produce both PI(3)P and PI(3,4)P2 species. Recent data suggests that platelet PI3K C2α controls a basal pool of PI(3)P with slow turnover rate that is not involved in agonist-induced PI(3)P generation or a major change in overall lipid composition [31]. These prior studies demonstrate that inactivation of platelet PI3K C2α results in a change in the composition of the spectrin-based-membrane skeleton, with measurable reductions in spectrin, myosin, filamin, moesin, GPIbα, and GPIIb content [31]. These changes in membrane skeleton composition were associated with an overall increase in plasma membrane rigidity and correlated defects in platelet thrombosis [31]. Our preliminary pharmacological data demonstrate that acto-myosin-based cytoskeletal dynamics are a key player in $\dot{\varepsilon}$-*S*, with inhibition of actin polymerization, turnover, and contractile function all leading to complete inhibition of $\dot{\varepsilon}$-*S* [Ca^2+^]_c_ flux. Taken together with the almost complete blockade of $\dot{\varepsilon}$-*S* [Ca^2+^]_c_ flux with MIPS-21335, these data point toward a mechanism whereby basal PI3K C2α activity and associated membrane [PI(3)P], in concert with membrane skeleton composition and dynamics, play a critical role in “coupling” externally applied $\dot{\varepsilon}$ to the $\dot{\varepsilon}$-*S* mechanosome and more specifically Piezo1 (Fig. 9). These findings track with the involvement of membrane bending rigidity or stiffness in modulating Piezo1 mechanosensing. A full delineation of the interplay between the membrane skeleton, PI3K C2α lipid products, and membrane dynamics while outside the scope of the current study is a subject for future investigation.

By mapping the precise temporal progression of platelet Ca^2+^ signaling to individual platelet trajectories in blood flow at idealized stenoses, we demonstrate that platelets exhibit distinct streamline-dependent Ca^2+^ signal activation statuses contingent on their acceleration profile. Critically, our data demonstrate that rather than responding to peak steadystate τ, platelet activation is initially triggered by very early transitions in free-flow strain at the very start of flow acceleration. Our experiments using isolated platelet perpetrations exposed to quasi-homogenous $\dot{\varepsilon}$ critically support this finding and demonstrate that platelet activation is attuned to the rate of change in $\dot{\varepsilon}$ application and On/Off in nature, such that $\dot{\varepsilon}$-*S* [Ca^2+^]_c_ flux shuts down upon immediate cessation of $\dot{\varepsilon}$. These findings track with our observations that platelet [Ca^2+^]_c_ flux at stenosis downregulates across the acceleration phase and continues to downregulate post-stenosis returning to baseline levels. This short-range effect suggests that the $\dot{\varepsilon}$-*S* mechanosome is triggered by acute changes in membrane and cytoskeletal elastic deformations and/or rapid changes in membrane tension that become quiescent once steadystate $\dot{\varepsilon\ }$is achieved. This concept is supported by our $\dot{\varepsilon}$ experiments where overall $\dot{\varepsilon}$-*S* [Ca^2+^]_c_ flux reduced by 50% when the rate of change in $\dot{\varepsilon}$ approached zero. While structural accommodations of the plasma membrane and underlying spectrin-based-membrane skeleton to the applied steadystate $\dot{\varepsilon}$ may in part explain this refractory behavior, voltage-dependent inactivation and desensitization of Piezo1 [41] and rapid desensitization of P2X1 [42] may also contribute to the observed decay of $\dot{\varepsilon}$-*S* [Ca^2+^]_c_ flux.

Early studies by Strony et al. [7] using experimental Folts and CFD models to correlate hemodynamics and platelet thrombosis raised the possibility that $\dot{\varepsilon}$ and elongational platelet deformation at stenosis entry are important in promoting thrombus formation. However, very few follow-up studies have investigated the impact of $\dot{\varepsilon}$ regimes on platelet activation. Based on computational modelling of a defined 84% axisymmetric arterial stenosis (in which the contraction and expansion in flow geometry followed a sinusoidal function), Bluestein et al. proposed that elongation stresses produced by convective acceleration at arterial contraction may play a role in platelet activation [43]. They reported that $\dot{\varepsilon}$, computed along a select near wall trajectory, reached values of up to 600 s^−1^ (at Re = 3600) [43]. These modelling data fall well within the range of the present study where we demonstrate that free-flow platelet activation can occur at an $\dot{\varepsilon}$ ≥747 s^−1^. In the case of the microcirculation, changes in vessel radius of ~20%, at a typical pre-constriction blood velocity of ~0.1 m/s, have been shown to generate $\dot{\varepsilon}$ of ~5 × 10^3^ s^−1^ [44]. Extensional strain of this magnitude, known to trigger unfolding of VWF and subsequent platelet adhesion, sits well within the midrange of our hyperbolic microfluidic studies (*Q* = 200 μL/min) which we show trigger significant adhesion-independent platelet [Ca^2+^]_c_ activation. In this context, $\dot{\varepsilon}$ regimes of the order of those utilized in this study may play a significant role in initial platelet activation under pathological conditions.

While taken together our experimental findings support our $\dot{\varepsilon}$-*S* mechanosome concept, there are some shortfalls in our experimental approach: (i) Chief among these is the reliance on a purely pharmacologic approach to investigate this phenomenon. This limitation is in part imposed by the rapidity of platelet functional responses under such acute velocity gradients and the intractability of human platelets to standard genetic manipulation. In addition, $\dot{\varepsilon}$-*S* [Ca^2+^]_c_ flux is triggered on a millisecond timescale making traditional approaches to the investigation of human platelet signal transduction difficult to employ. In future studies, we aim to utilize available transgenic mouse models to further explore the key platelet signaling regulators of $\dot{\varepsilon}$-*S*, with a specific focus on the way in which PI3K C2α regulates this mechanotransduction process. However, it must be born in mind that due to the relative difference in size of human versus mouse platelets the hemodynamic conditions required to trigger $\dot{\varepsilon}$-*S* [Ca^2+^]_c_ flux may be substantially different; (ii) Our use of time-averaged confocal imaging data to investigate very rapid platelet signaling under very high velocity flow is based on a compromise between Ca^2+^ probe (CAL520) signal strength (due to quantum yield and dye loading efficiency) and the need to derive high-speed data on platelet activation. This is in part due to the frame rate limitations of the resonant scanning system employed. By integrating CFD-based platelet trajectory modelling with time-averaged imaging data, we were able to gain access to the millisecond change in platelet Ca^2+^ signaling. While powerful, this approach only enables interrogation of the ensemble average of platelet activation along predicted flow trajectories and does not enable the monitoring of single platelet responses. This is primarily a hardware limitation and may be overcome in the future through the use of ultrafast μ-PIV-based imaging techniques; (iii) The generalized blood density and viscosity CFD model employed, while capable of accurately predicting overall platelet behavior within our model system(s), is not a non-Newtonian model per se and therefore does not consider the inertial effects of red blood cell and platelet mass on overall blood flow behavior; (iv) Finally, our conclusions are based on a purely synthetic reductionist approach utilizing microfluidic geometries to impose idealized supraphysiological hemodynamic gradients on blood components. In future studies, we aim to expand on this analysis and extract and model hemodynamic conditions found at pathological stenosis and within MCS pumping systems to investigate a role for $\dot{\varepsilon}$-*S* in the pathology associated with these empirical systems.

## Conclusions

In conclusion, our working hypothesis is that $\dot{\varepsilon}$-*S* represents the very earliest event in platelet activation under supraphysiological blood flow hemodynamics. In the context of overall platelet activation and aggregation, we posit that $\dot{\varepsilon}$-*S* acts as a “priming mechanism” in response to initial flow acceleration, initiating platelet activation in free flow, allowing platelets to rapidly form immediate downstream adhesive interactions under the limiting effects of high relative flow velocities. This working model does not rule out a coincident role for $\dot{\varepsilon}$ during flow acceleration in promoting VWF unfolding [17] nor does it exclude a role for extreme $\dot{\gamma}$ (>30,000 s^−1^) in promoting concomitant VWF self-association and platelet aggregation [45], but rather posits that $\dot{\varepsilon}$-*S* is coincident with these VWF-dependent processes and takes on an increasingly dominant role as hemodynamic gradients become more severe.

## Methods

### Symmetrical stepped stenosis microfluidic design

Stepped stenosis microchannel geometries were adapted from [46]. Symmetrical stenosis microfluidics (allowing for duplicate assay) were designed with entry and exit lengths of 6 mm and channel width of 600 μm; allowing for full flow development prior to stenosis (Additional file 1: Fig. S1A & B). A 30-μm pillar array filter prior to the primary channel prevents debris reaching the symmetrical stenoses, which protrude 280 μm into the channel, with a gap distance between mirror image stenoses of 40 μm and gap length of 20 μm (Additional file 1: Fig. S1B). Entry angle (*θ*_e_) variations (internal angles 20, 40, 60, and 80°) were generated keeping all other geometric parameters constant. Microfluidic arrays consisting of 14 independent channels per chip were fabricated allowing for high-throughput imaging and assay (Additional file 1: Fig. S1A). The entire microfluidic array was vacuum bonded to an underlying No. 1 borosilicate microscope coverslip via an integrated vacuum chuck allowing for easy reuse and coverslip changeover (Additional file 1: Fig. S1A). Hemodynamic control of the 14 independent on-chip microchannels was controlled by a custom built Tecan cavro centris syringe pump manifold (Tecan Group AG, Switzerland) allowing for serial or parallel perfusion experiments, sample management, and microchannel purging. Overall sample flow rates and sample switching control was driven by custom Python script.

### Computational fluid dynamics—stepped stenosis microfluidics

3D CAD models of the stepped stenosis geometries on-chip were prepared using SolidWorks® (Dassault Systèmes, Vélizy-Villacoublay, France). Models were meshed with a mixture of tetrahedral and hexahedral unstructured elements (Pointwise, Inc. Fort Worth, Texas, USA) for CFD simulations. The total number of mesh elements ranged from 2.8 to 4.8 million. Mesh-independent studies were carried out for an index case to ensure adequate mesh resolutions. Finer mesh elements were used near the wall to ensure adequate resolution of the higher spatial velocity gradients there. CFD calculations were performed according to [47] with suitable modifications to the boundary conditions and a generalized blood density and viscosity model to accommodate the difference between coronary flow and present microfluidic devices. In brief, an in-house developed CFD solver based on OpenFOAM (v7, The OpenFOAM Foundation Ltd., UK) was used to simulate blood flow within each device [48]. A constant inflow, with matching flow rate at 200 μL/min, was applied to the “INLET” (Additional file 1: Fig. S1A) of the device model. The velocity profile was allowed to fully develop with an extended model length upstream of the stenosis. Constant pressure was applied at the “Outlet” of the model. All simulations were performed for at least 1 s (i.e., ~10 washouts) to flush out any initial transient effect. Velocity, local $\dot{\gamma}$ and *τ* were extracted from tracing of individual (massless) particle paths. A total of 59 tracings (at resolutions of 1 μm [near wall] to ~300 μm [centerline]) were computed (Fig. 1B). After an initial analyses, 3 subsamples (T1, T4, and T14) were reported to showcase critical behaviors (Fig. 1C–H).

### Hyperbolic device design and assay

A hyperbolic-shaped microchannel geometry was used to examine the effect of strain/stress loading on human platelets under spatially uniform extensional strain rate [49]. Hyperbolic contraction channel microfluidics were designed with contraction length (*L*_*h*_) = 300 μm + (*L*_*g*_) = 20 μm; contracting from a straight channel width (*Wu*) = 600 μm to (*Wc*) = 40 μm (Additional file 1: Fig. S4). At the end of the hyperbolic contraction, a sudden expansion (*W*_*d*_) = 600 μm was used to investigate platelet [Ca^2+^]c flux recovery after the release of the fluidic force (Additional file 1: Fig. S4). All hyperbolic channel replicas had a constant height of *z* = 80 μm. Channels were incubated for 10 min with 2% w/v Pluronic F127 (Sigma Aldrich) and BSA (10%w/v) prior to platelet perfusion to prevent cell adhesion. Isolated human platelets were suspended in modified Tyrodes buffer (pH7.2) containing CaCl_2_ (1 mM), MgCl_2_ (1 mM), and 0.5%w/v methyl cellulose (Sigma Aldrich); final density = 1016 kg m^−3^ and viscosity of 0.004 ± 0.015 Pa.s [50]. Platelet [Ca^2+^]_c_ dynamics were acquired for a duration of 30 s at a frame rate of 0.067 fps via resonant scanning confocal imaging (Nikon Plan Fluor × 40 WI/0.50 objective) using an Andor Zyla sCMOS camera (image scan size of 512 × 512), with a focal point at *z* = 40 μm. Fluorescence image analysis was restricted to a 4-μm-wide line scan running along the central axis of the microfluidic, representing 0.1 × *W*_*c*_ at which $\dot{\varepsilon}$ is considered uniform. Under these conditions, the hyperbolic region produces a uniform Hencky strain [51] *ε*_*H*_=2.7, i.e.,

1 $${\varepsilon}_H=\mathit{\ln}\left(\frac{w}{w_c}\right)$$

and Cauchy strain rate ($\dot{\varepsilon}$), based on the assumptions of; ideal hyperbolic flow, a spatially uniform viscosity of 0.004 Pa.s, channel height *z* = 80 μm, and *Q* = 12.5, 50, 200, and 600 μL/min, of $\dot{\varepsilon}=$ ~318 s^−1^, ~1309 s^−1^, ~5458 s^−1^, and ~16,013 s^−1^_,_ i.e.,

2 $$\dot{\varepsilon}=\frac{Q}{L_ch}\left(\frac{kc}{w_c}-\frac{kd}{w}\right)$$

Based on the same assumptions, the flow extensional stresses *σ*_*E*_ = 1.3 Pa, 5.3 Pa, 21.8 Pa, and 64.1 Pa, respectively.

### Computational fluid dynamics—hyperbolic microfluidic

Solid Edge (Siemens PLM Software, USA) was used to extract the simulation domain from the 3D CAD model of the hyperbolic microfluidic channel. A hexahedral unstructured mesh with 2.3 million elements was generated using Pointwise, Inc. (Fort Worth, Texas, USA). Finer mesh elements were used around the domain’s centerline in the stenosis and downstream of it to investigate the impact of the hyperbolic geometry on the extensional strain rate and its gradient. A mesh convergence study was carried out to ensure adequate mesh resolution. The flow was assumed to be laminar, incompressible, and Newtonian with a viscosity of 0.004 Pa.s. The considered governing equations for conservation of mass and momentum were as per [49]. Numerical simulation of the flow through the hyperbolic channel was performed using OpenFOAM v8 (The OpenFOAM Foundation Ltd., UK). The steadystate solver for incompressible flow simpleFoam, which is based on the SIMPLE (Semi-Implicit Method for Pressure Linked Equations) algorithm, was chosen for the simulations. A constant uniform inflow was applied at the “Inlet” of the model, the model length upstream was sufficient to allow for fully developed flow in the hyperbolic segment. Constant pressure was applied at the “Outlet” of the model. No-slip condition at the solid walls was imposed. The simulations were performed for four different inflow rates: *Q* = 12.5 μL/min, 50 μL/min, 200 μL/min, and 600 μL/min. An open-source, multi-platform data analysis and visualization application ParaView 5.6.0 (Sandia National Laboratories, Kitware Inc, Los Alamos National Laboratory) was used for visualization and extraction of the streamwise velocity along the centerline of the model. Python code was used to calculate the extensional strain rate and its gradient for each of the considered cases.

### Microfluidic fabrication

All microfluidic devices were fabricated using established soft photolithography methods [46]. Briefly, using maskless lithography (MLA 150, Heidelberg instruments), a master template was patterned onto a 4-in. silicon wafer using SU-8 3050 photoresist (MicroChem Corp.) to produce defined channel features with a height of 100 μm for the symmetric step and 80 μm for the hyperbolic geometry. PDMS (Sylgard 184) mixed with a curing agent in a 1:10 ratio by weight, degassed in a vaccum dessicator, and cast on the master template and cured at 130 °C in a convection oven for 15 min. The cured PDMS was peeled off the mold, cut to size, and punched with a 0.75-mm hole at the inlets, outlets, and vacuum chuck, before being reversibly sealed onto a No. 1 borosilicate microscope coverslip. Microfluidic chips were re-cycled between use via sonication in 20% Extran MA05 (Merck Millipore), followed by sonication in DI water, and baked in a convection oven at 110°C following a final DI rinse.

### Blood collection and handling

Ethics approval was obtained from Monash University Human Research Ethics Committee. Blood from healthy consenting volunteers was withdrawn using a 19G butterfly needle into syringes containing either acid citrate dextrose (ACD) [ratio of 6:1 (blood/ACD)] for platelet and red blood cell isolation or 800 U/mL Lepirudin© for VWF aggregation assay and FACS analysis. Samples were gently inverted to mix anticoagulant, transferred to 50-mL Falcon® tubes, and allowed to rest for 10 min at 37 °C prior to use.

### Platelet isolation

Platelet isolation from human whole blood was carried out as per [52]. Briefly, human whole blood collected in ACD was supplemented with 0.005 U/mL apyrase before incubation at 37 °C for 10 min. PRP was isolated by centrifugation at 200×*g* for 15 min, followed by another centrifugation at 1700×*g* for 7 min, where platelet poor plasma (PPP) was removed and platelet pellet resuspended in an equal volume of platelet washing buffer (PWB) [4.3 mM K_2_HPO_4_, 4.3 mM Na_2_HPO_4_, 24.3 mM NaH_2_PO_4_, 113 mM NaCl, 5.5 mM D-glucose, and 10 mM theophylline, (pH 6.5) containing 0.5% w/v BSA, 0.01 U/mL apyrase, and 800 U/mL hirudin]. A final centrifugation was performed on the platelet suspension at 1500×*g* for 7 min and the platelet pellet was resuspended in PWB (supplemented with 0.02 U/mL apyrase) to achieve a platelet concentration of 300 × 10^9^/L. For CellTracker Green (CMFDA) experiments, isolated platelets were rested at 37 °C for 30 min with 10 μM CMFDA. Following the incubation period, CMFDA-labelled platelets were reconstituted with washed RBC (40% final hematocrit, Hct) prior to perfusion through the device (Fig. S2B).

### Platelet Ca^2+^ (CAL520) assay

Platelet calcium assay was adapted from published methods [52, 53]. Washed platelets (300 × 10^9^/L) were loaded with 1.25 μM CAL520 (2-aminophenoxy)ethane-N,N,N′,N′-tetraacetic acid tetra(acetoxymethyl)ester (JOMAR Life Research) for 45 min at 37 °C. CAL520-loaded platelets in Tyrodes + 1mM Ca^2+^/Mg^2+^ were then reconstituted with washed RBC (40% final hematocrit; Hct) prior to perfusion through stepped stenosis microfluidics. Hct and platelet counts were performed by full blood analysis using Cell-Dyn Emerald® hematology analyzer (Abbott Diagnostics). [Ca^2+^]_c_ dynamics were acquired for a duration of 30 s with a “no delay” interval for highest speed via resonant scanning confocal imaging (Nikon Plan Fluor × 40 WI/0.50 objective) using an Andor Zyla sCMOS camera at 15 frames per second (image scan size of 512 × 512). All image analysis was performed off-line in FIJI. The corrected fluorescence values (F) for each region of interest (ROI) or line scan profile were converted into pseudo-ratio values according to:

3 $${\left[{\mathrm{Ca}}^{2+}\right]}_{\mathrm{c}}={\mathrm{K}}_{\mathrm{d}}\ \left[\left(\mathrm{F}-{\mathrm{F}}_{\mathrm{min}}\right)/\left(\mathrm{F}-{\mathrm{F}}_{\mathrm{max}}\right)\right]\ \left[{\mathrm{K}}_{\mathrm{d}}\ \mathrm{CAL}520=320\ \mathrm{nM}\right]$$

where *F*_min_ was derived for independent platelet flow experiments in which CAL520-loaded platelets were treated with 50 μM DM-BAPTA-AM (Sigma Aldrich) and resuspended in Tyrodes buffer supplemented with 5 mM EGTA and *F*_max_ derived for independent platelet flow experiments in which CAL520-loaded platelets were treated with 2.5 μM A23187 (Sigma Aldrich) and resuspended in Tyrodes buffer supplemented with 5 mM CaCl_2_).

### CFD-directed Ca^2+^ image analysis

Image analyses were performed off-line using a custom macro in FIJI (ImageJ). To analyze trajectory-dependent changes in platelet [Ca^2+^]_c_ flux, resonant scanning confocal image timelapse stacks were imported into FIJI using Bio-Formats. Stacks were background corrected and a *z*-projection (sum slices) generated to create a time-averaged image (304 total frames). *Z*-projections were thresholded using Huangs fuzzy logic (Light). CFD-derived platelet trajectories were plotted in FIJI as concatenated *x*,*y* coordinates using the makeLine (*x*1, *y*1, *x*2, *y*2, lineWidth) macro (line width = 5 μm) and imported into the FIJI ROI manager. Trajectories were overlaid on *z*-projections from ROI manager. To align trajectories three fiducial markers located at the base and top of the microfluidic features were included in the ROI set and manually aligned with the imaging data. Multiplot line scan profiles using the CFD-defined trajectories were analyzed and intensity data extracted. This process was carried out for *F*, *F*_min_, and *F*_max_ data sets and [Ca^2+^] along profile scans assessed. To gain a statistically significant sample size across multiple blood/platelet donors, a ROI subset was assessed for select CFD trajectories for each donor. Then, 10-μm-diameter ROIs were defined by their *x*,*y* coordinate positions equivalent to the start of acceleration (Acc1), the midpoint of acceleration (Acc2), the endpoint of acceleration adjacent to the stenosis apex (Acc3), the midpoint of stenosis apex (Apex), and reference points positioned 3 mm up and downstream of the apex midpoint were overlaid on the *z*-projected data sets. Fluorescence intensities for each ROI were determined for each using the Analyze/measure function in FIJI. This ROI-dependent analysis was performed for each ROI across *F*, *F*_min_, and *F*_max_ samples and [Ca^2+^] determined. The analysis Macro, CFD trajectory, and ROI coordinates are available on request.

### Red blood cell isolation

RBC isolation was carried out as per [52]. Briefly, human whole blood was collected in ACD and rested at 37 °C for 10 min. Apyrase (0.005 U/mL) was added to the blood to remove any ADP secreted during venipuncture, and centrifuged at 200×*g* for 15 min. The platelet-rich plasma (PRP) supernatant along with the buffy coat was carefully discarded by aspiration. The remaining packed RBC were washed 3× with an equal volume of 1× Tyrode’s buffer [10 mM Hepes, 12 mM NaHCO_3_, 137 mM NaCl, 2.7 mM KCl, and 5 mM glucose (pH 7.4)] and mixed by gentle inversion and subsequently centrifuged at 1700×*g* for 7 min (50 mL) to pack the erythrocyte phase.

Where fixed RBC were required, packed RBC were resuspended in an equal volume of 0.16%v/v glutaraldehyde in 1 × Tyrodes (1:1v/v) to a final concentration of 0.08%v/v and incubated for 10 min at room temperature. Fixed RBC were subsequently washed 5× (as above) with resuspension in an equal volume of supplemented 1× Tyrode’s buffer (+1 mM CaCl_2_ + 0.5 % w/v BSA). The final packed RBC suspension was supplemented with 800 U/mL hirudin and 0.02 U/mL apyrase and allowed to rest for 30 min at 37 °C. Immediately prior to reconstitution with isolated platelets, the RBC suspension was supplemented with a further 0.02 U/mL apyrase to prevent erythrocyte derived ADP from activating the isolated platelet suspension.

### VWF microfluidic aggregation assay

Platelet aggregation was performed as previously described [46]. Microchannels were selectively derivatized with human VWF (10 μg/ml; isolated from Biostate CSL Ltd) for 10 min, such that only the peak stenosis and downstream stenosis face were coated, to allow for focal platelet capture and adhesion to the PDMS surface (Fig. S1C & D). VWF was manually injected into the channel outlet (Fig. S1A) to coat downstream up until the apex of the stenosis geometry. Following 10-min incubation, VWF was aspirated from the outlet (Fig. S1A) to remove any excess and the PDMS was vacuum sealed onto a new isopropanol-washed coverslip. The channels were then subsequently blocked with 2% w/v BSA via the channel inlet for 10 min to passivate the non-VWF-coated upstream regions, prior to wash out with unmodified tyrodes buffer pH 7.4. Hirudinated human whole blood was incubated with the lipophilic membrane dye DiOC_6_ (1 μg/mL) for 10 min at 37 °C prior to device perfusion. Where drug treatments were required, blood samples were treated with the reagent and incubated for 10 min at 37 °C prior to device perfusion. Platelet aggregation at the stenosis was monitored via epifluorescence at 1 fps for 180 s (Nikon Ti-E microscope Plan Fluor × 20/0.50 objective with Andor Zyla sCMOS detector). Aggregate size was assessed as per [46] and expressed as mid-plane surface area in square micrometers.

### FACS analyses

Prior to perfusion, microfluidic devices were coated with 2% w/v BSA for 10 min prior to wash out with unmodified tyrodes buffer pH 7.4. Hirudin anticoagulated whole blood was either perfused through the external syringe pump line as a baseline control (no microfluidic perfusion) or *θ*_e_ = 80° geometry at *Q* = 200 μL/min, or no perfusion as a resting control. Each sample was diluted 1:10 in 1× Tyrode’s buffer (+1 mM CaCl_2_ + 0.5 % BSAw/v) and 5 μL of the diluted sample was immediately transferred to their respective Eppendorf tubes containing α−CD42bAPC (Becton Dickinson), and either 5 μL PAC-1 FITC (Becton Dickinson) or 5 μL α−P-selectin-PE (Life Technologies) . The tube containing positive control was activated with 40 μM ADP and all samples were let to rest for 15 min. Reaction was stopped by adding 600 μL 1× Tyrode’s buffer and immediately transferred to 12 × 75 mm Falcon® polystyrene test tubes, and all samples were assessed within a 2-h window. Platelets were identified by light scatter characteristics and confirmed by CD42b expression. Positive controls were used to adjust voltages. Acquisition was performed on a BD FACSCanto-II (Becton Dickinson) that was routinely calibrated with Calibrite beads in conjunction with FACSComp Version 5.1 software (Becton Dickinson). The results were analyzed with FlowLogic 7.2.1 flow cytometric analysis software.

### Statistical analysis

Two-way ANOVA and Tukey’s multiple comparisons test were performed in GraphPad Prism. Nonlinear curve fitting of microfluidic aggregation experiments was fitted by least squares regression using an [Agonist] vs. response Variable slope (four parameters; no weighting) model with the Bottom constrained to a constant = 0. Where indicated statistical significance is defined as **** *P* < 0.0001; *** *P* = 0.0001 to 0.001; ** *P* = 0.001 to 0.01; * *P* = 0.01 to 0.05; ns *P* ≥ 0.05.

## Supplementary Information

**Additional file 1: Fig S1.** High-throughput stenosis microfluidics platform & platelet aggregation assay method schema. **Fig S2.** Ca^2+^ trajectory sampling and hemodynamics. **Fig S3.** Effect of stenosis entry geometry on trajectory dependent hemodynamics and platelet function. **Fig S4.** Hyperbolic microfluidic geometry & platelet [Ca^2+^]_c_ assay schema. **Fig S5.** Effect of NF449 and Cbx on platelet aggregation.

## Data Availability

The datasets supporting the conclusions of this article are included within the article (and its supplementary files).
