# Supplementary material for: An extensional strain sensing mechanosome drives adhesion-independent platelet activation at supraphysiological hemodynamic gradients
Source: BMC Biol. 2022 Mar 24;20:73. doi: 10.1186/s12915-022-01274-7 (PMC8944166; doi:10.1186/s12915-022-01274-7)
Supplement: Supplementary file 1 — Additional file 1: Fig S1. High-throughput stenosis microfluidics platform & platelet aggregation assay method schema. Fig S2. Ca2+ trajectory sampling and hemodynamics. Fig S3. Effect of stenosis entry geometry on trajectory dependent hemodynamics and platelet function. Fig S4. Hyperbolic microfluidic geometry & platelet [Ca2+]c assay schema. Fig S5. Effect of NF449 and Cbx on platelet aggregation. [file 12915_2022_1274_MOESM1_ESM.docx]

**Additional File 1**

An extensional strain sensing mechanosome drives adhesion-independent platelet activation at supraphysiological hemodynamic gradients

**Authors:** Nurul A. Z. Abidin^1^, Eric K. W. Poon^2^, Crispin Szydzik^1^, Mariia Timofeeva^3^, Farzan Akbaridoust^3^, Rose J. Brazilek^1^, Francisco J. Tovar Lopez^4^, Xiao Ma^5^, Chitrarth Lav^3^, Ivan Marusic^3^, Philip E. Thompson^5^, Arnan Mitchell^4^, Andrew S. H. Ooi^3^, Justin R. Hamilton^1^, Warwick S. Nesbitt^1^*

Warwick S. Nesbitt, PhD

Email: [warwick.nesbitt@monash.edu](mailto:warwick.nesbitt@monash.edu)

**This PDF file includes:**

Figures S1 to S5


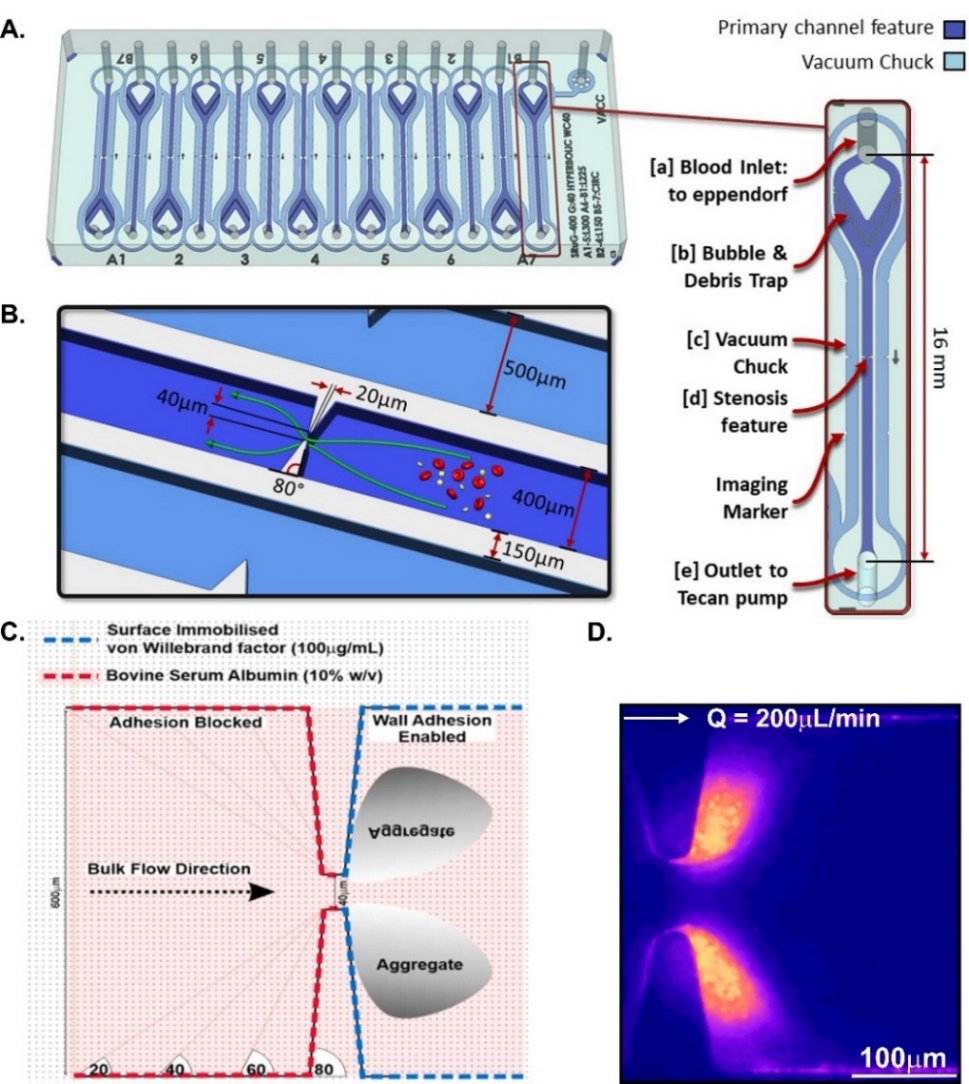
Fig. S1. High-throughput stenosis microfluidics platform & platelet aggregation assay method schema.

**(A)** Schematic of the high-throughput stenosis microfluidic chip showing a macro-view of the 14-channel array and configuration. Note that the chip was bonded to a No. 1 borosilicate microscope coverslip via the integrated vacuum chuck, with negative pressure controlled simply using an attached syringe housed in a custom-built jig. (**Inset**) Zoomed view of a single microfluidic unit showing: [**a**] the blood inlet port; [**b**] V-shaped post-array debris trap **[c]** vacuum chuck (to create a stable high-pressure seal to the underlying coverslip); **[d]** stenosis geometry; **[e]** downstream outlet connecting to multi-channel Tecan syringe driver. Note that the chips were driven using a custom-built multi-channel Tecan pump manifold controlled via Python script allowing for automated microfluidic channel switching and perfusion, flow rate control, buffer-blood exchange, and downstream blood sampling (FACS assay). (**B)** Schematic of the θ_e_ = 80^o^ symmetrical stepped stenosis geometry on-chip. (**C)** Schema describing the microfluidic platelet aggregation assay (see Methods). Purified von Willebrand factor (VWF - 100μg/mL) was manually perfused into the stenosis microfluidic via the downstream outlet port [**e**]. VWF was perfused to the downstream face of the stenosis geometries such that only the downstream face was coated – blue dotted region. The VWF sample was allowed to interact with the polydimethylsiloxane (PDMS) surface for 10 min and then subsequently aspirated via the downstream outlet port [**e**]. The vacuum was released and the PDMS chip was subsequently pealed from the underlying cover-glass, dried under N_2_ stream, and adhered to a clean coverslip. The entire microfluidic system was subsequently perfused with 10%w/v Bovine Serum Albumin (BSA) via the upstream blood inlet port [**a**] to block all naive PDMS and the underlying coverslip. This step specifically blocked the upstream face and apex of the PDMS stenosis geometry – red dotted regions. Following incubation with BSA for 10 min the microfluidic was flushed with 1x Tyrodes buffer pH 7.2 prior to blood perfusion experiments. This process created a VWF derivatized system such that only the downstream PDMS face of the stenosis geometry was competent for platelet adhesion and aggregation. In addition, this process blocked all platelet adhesive interactions within the acceleration in-flow component of the stenosis geometries, such that any effects of changing acceleration geometry were due to free-flow parameters and not platelet adhesion events. (**D)** Representative epifluorescence microscopy image of DiOC_6_ (1μg/mL) labelled human whole blood undergoing site directed aggregation within the θ_e_ = 80^o^ microfluidic stenosis geometry.

**
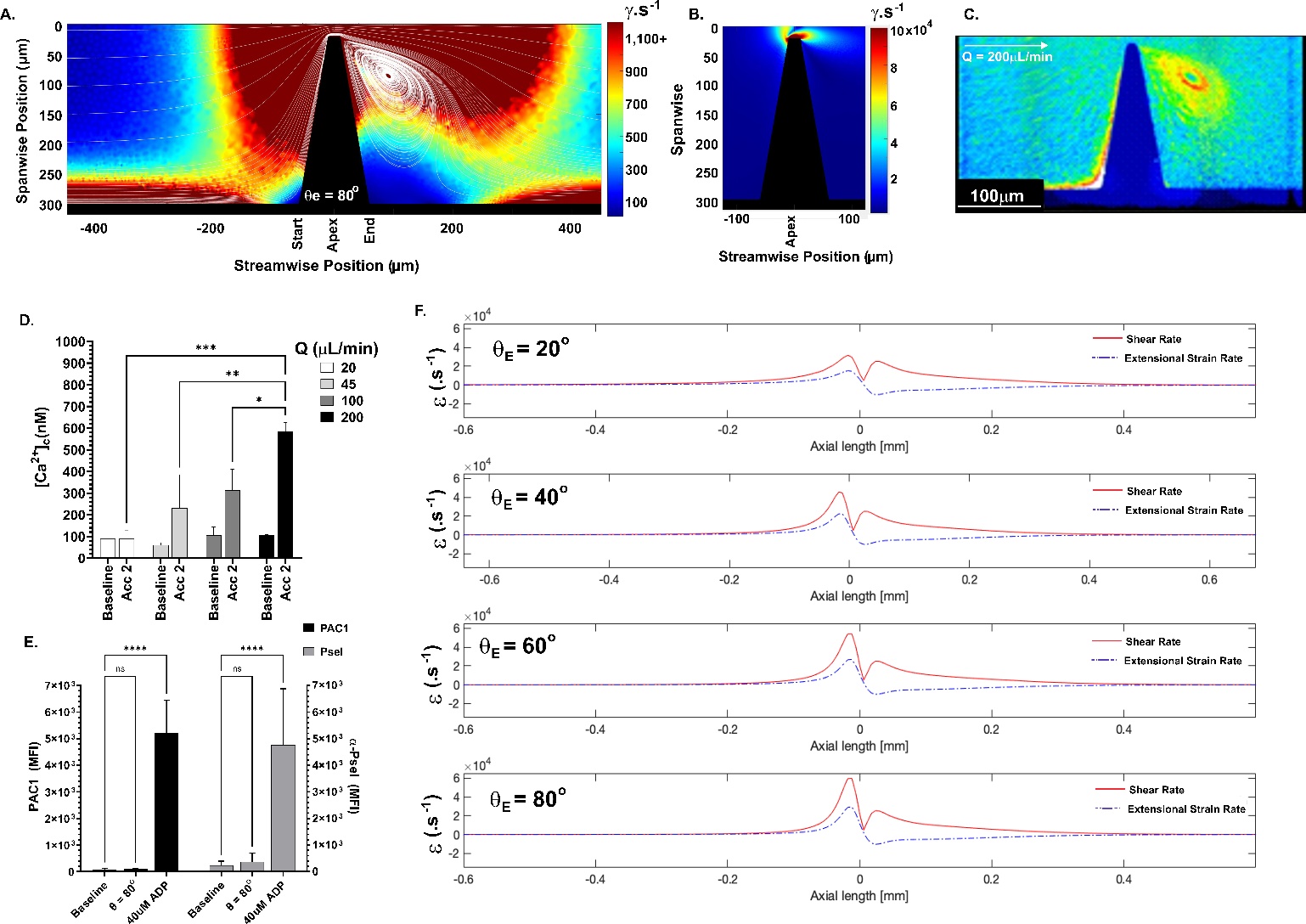
Fig. S2. Ca^2+^ trajectory sampling and hemodynamics.**

**(A)** Color map showing strain rate distributions within an x,y plane 50μm from the microchannel floor (derived from CFD modelling of non-Newtonian blood flow; Q = 200μL/min) at a prototypical symetrical stenosis with θ_e_ = 80^o^. White lines on the strain map show all 59 computed trajectories for particle release. **(B)** Modified color map showing strain rate distributions at stenosis apex within an x,y plane 50μm from the microchannel floor (derived from CFD modelling of non-Newtonian blood flow; Q = 200μL/min) at a prototypical symmetrical stenosis with θ_e_ = 80^o^. (**C)** Maximum projection confocal micrograph showing time averaged imaging of CytoTracker© Green (CMFDA) labeled human platelets reconstituted with Tyrodes + RBC (Hct = 40%) under perfusion through θ_e_ = 80^o^ stenosis geometry at Q = 200μL/min. Note that coordinates of observed vortices and platelet trajectory streamlines closely match those predicted by CFD particle release analysis (**D)** Ca^2+^ trajectory (T14) sampling following perfusion of reconstituted blood samples through θ_e_ = 80^o^ stenosis geometry at flow rates (Q) = 20, 45, 100, and 200 μL/min (N = 3 independent experiments). Note that [Ca^2+^]_c_ scales as a function of Q. (**E)** Whole blood FACS analysis showing platelet integrin α_IIb_β_3_ activation (Pac-1 binding) and surface P-selectin expression as a function of stenosis entry geometry: Baseline – endpoint sampling (no microfluidic perfusion); ADP (40μM) +ve control; θ_e_ = 80^o^ – perfusion through 80^o^ stenosis geometry (Q = 200μl/min). Note that blood sampling occurred 6mm from stenosis apex and correlates with platelet [Ca^2+^]_c_ measurements 3mm post-apex, with no significant surface expression over Baseline controls (N = 3 independent experiments). **(F)** CFD modelling of non-Newtonian blood flow (Q = 200μL/min) showing overall shear rate profiles and overall Extensional Strain rate profiles for θ_e_ = 20^o^, 40^o^, 60^o^, and 80^o^ stepped microfluidic geometries.

**
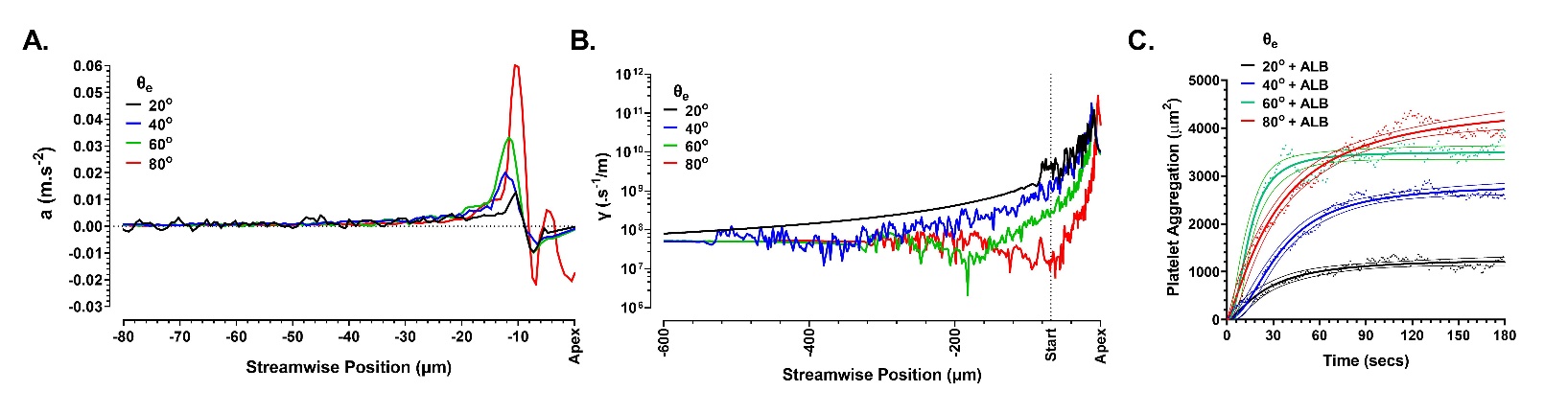
Fig. S3. Effect of stenosis entry geometry on trajectory dependent hemodynamics and platelet function.**

**(A)** Predicted change in acceleration for T14 as a function of θ_e_. (**B)** Predicted $\dot{\gamma}-$gradient profiles for T14 as a function of θ_e_. **C.** Platelet aggregation at stenosis apex as a function of θ_e_ following treatment of DiOC_6_ labelled human whole blood with amplification loop blockers (ALB) for 10 min: Indomethacin (10μM) to inhibit COX1; MRS2179 (100μM) to inhibit P2Y1; 2-MeSAMP (10μM) to inhibit P2Y12 ADP dependent signaling. Curves shown are [Agonist] vs. response Variable slope (four parameters) Least squares fit + 95% CI of N= 3 - 5 independent experiments. Note, the stepwise reduction in platelet aggregation as a function of θ_e_ in the absence of platelet amplification loops.

**
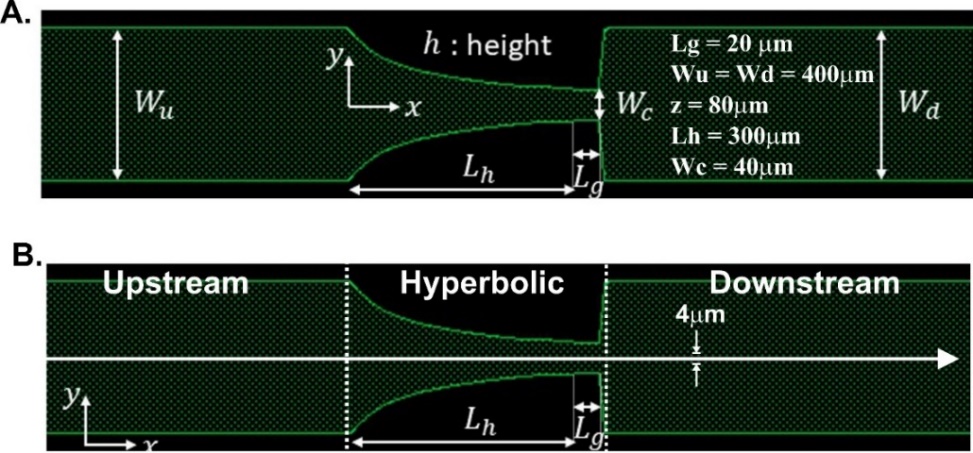
**

**Fig. S4. Hyperbolic microfluidic geometry & platelet [Ca^2+^]_c_ assay schema.**

**(A)** Schematic of the Hyperbolic microfluidic developed to apply uniform quasi-homogenous extensional stress/strain as a function of flow rate to isolated platelet samples. (**B)** Schema showing line scan sampling region for image analysis categorized into Upstream, Hyperbolic, and Downstream zones. Note that platelet [Ca^2+^]_c_ was assessed as a function of location within the hyperbolic microfluidic with a 4μm (equivalent to 0.1x*W_c_*) wide central flow streamline representing the region of quasi-homogenous extensional strain within the device.

**Fig. S5. Effect of NF449 and Cbx on platelet aggregation.**


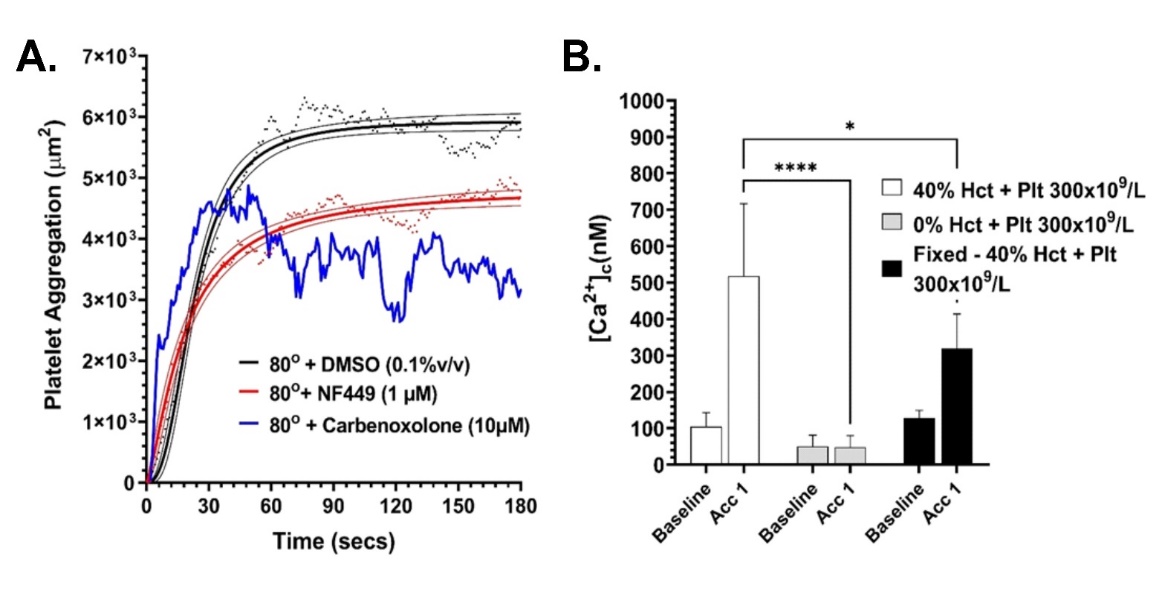


Platelet aggregation at stenosis apex θ_e_ = 80^o^ following treatment of DiOC_6_ labelled human whole blood with, DMSO (0.1%v/v); NF449 (1μM); Carbenoxolone (10μM). Curves shown are [Agonist] vs. response Variable slope (four parameters) Least squares fit + 95% CI of N= 3 - 5 independent experiments.
